# Molecular features of prenylated (iso)flavonoids from Fabaceae in relation to their potential NorA inhibition in *Staphylococcus aureus*

**DOI:** 10.3389/fphar.2025.1715533

**Published:** 2026-01-30

**Authors:** Marina Ika Irianti, Janniek Henrieke Ritsema, Jos Hageman, Jean-Paul Vincken, Carla Araya-Cloutier

**Affiliations:** 1 Laboratory of Food Chemistry, Wageningen University and Research, Wageningen, Netherlands; 2 Laboratory of Microbiology and Biotechnology, Faculty of Pharmacy, Universitas Indonesia, Depok, Indonesia; 3 Biometris, Applied Statistics, Wageningen University and Research, Wageningen, Netherlands

**Keywords:** efflux pump inhibitors, Fabaceae, fluoroquinolone, NorA, prenylated (iso)flavonoids, QSAR, *Staphylococcus aureus*

## Abstract

**Background:**

The overexpression of the NorA efflux pump is known to be an important factor in the antimicrobial resistance mechanism of *Staphylococcus aureus*. Therefore, NorA inhibition can help disarm this pathogen and tackle antimicrobial resistance. In this study, we aim to unravel the molecular properties of prenylated (iso)flavonoids from Fabaceae as potential NorA inhibitors and to propose leading compounds for future research.

**Methods:**

A collection of 37 prenylated isoflavonoids and flavonoids (obtained by purification, chemically synthesized, or commercially purchased), collectively referred to as (iso)flavonoids, was evaluated for its activity against *norA*-overexpressing *Staphylococcus aureus* using the checkerboard assay with ciprofloxacin (NorA substrate) and erythromycin (primarily non-NorA substrate), in combination with ethidium accumulation assays. Moreover, a *norA*-knockout *Staphylococcus aureus* strain was used to corroborate the specificity of the observed effects. Subsequently, *in silico* binary QSAR and pharmacophore models were developed to elucidate the key molecular properties for potential NorA inhibition.

**Results:**

Seven prenylated (iso)flavonoids, namely, 8-prenylnaringenin, 6-*C*,7-*O*-diprenylnaringenin, glabrene, neobavaisoflavone, wighteone, licoisoflavone A, and glycyrrhisoflavone, potentiated ciprofloxacin up to 8-fold in *norA-*overexpressing and up to 2-fold in *norA-*knockout strains at 10 μM, without any membrane permeabilization effects. Moreover, prenylated (iso)flavonoids potentiated erythromycin in *norA-*overexpressing *Staphylococcus aureus* only up to 2-fold. Binary QSAR models were generated using datasets from the checkerboard and ethidium accumulation assays with a total prediction accuracy of up to 90% for active and 88% for inactive compounds. Based on QSAR models, the polar surface area, the balance of hydrophobicity and hydrophilicity, and the overall hydrophobicity were correlated with antibiotic potentiation and efflux inhibition of prenylated (iso)flavonoids. Moreover, in our study, we revealed that fractional negative polar surface area and formal (negative) charges are key properties that differentiate prenylated (iso)flavonoids with antimicrobial activity from those that act as potential NorA inhibitors. A pharmacophore model provided the basis for further optimization of prenylated (iso)flavonoids, mainly neobavaisoflavone and wighteone, as potential NorA inhibitors.

**Conclusion:**

In our study, we provide, for the first time, predictive QSAR models of prenylated (iso)flavonoids as potential NorA inhibitors and propose two potential leads based on this family of plant-derived compounds. Future research on the specificity and validation of prenylated (iso)flavonoids as NorA inhibitors is required.

## 1 Introduction

*Staphylococcus* *aureus* (*S. aureus*) is one of the most pathogenic Gram-positive bacteria responsible for infections in hospitals, community settings, and livestock farming (Silva et al., 2023). Methicillin-resistant *S. aureus* (MRSA) has become the most prevalent strain worldwide (Guo et al., 2020), and emerging resistance to fluoroquinolones in this strain is increasing (Alseqely et al., 2021). Unfortunately, the introduction of a new antibiotic to replace fluoroquinolones is often followed by the development of antimicrobial resistance (AMR) (Li et al., 2025). Therefore, discovering resistance-modifying agents (RMAs) to increase the potency of new and traditional antibiotics has become urgent (Kumar et al., 2023). AMR in *S. aureus* can be mediated by the presence of the NorA efflux pump from the major facilitator superfamily (MFS) (Yu et al., 2022). The *norA* efflux pump gene was found to be overexpressed in 43% of resistant *S. aureus* strains (Patel et al., 2010; Dashtbani-Roozbehani and Brown, 2021). Discovering efflux pump inhibitors (EPIs) against NorA can help tackle the resistance of *S. aureus* and restore the activity of existing antibiotics.

The Fabaceae family is listed as one of the largest plant families that contain more than 490 medicinal plant species (Abdelsalam et al., 2022). This plant family contains important secondary metabolites, such as phenolics, alkaloids, peptides, and terpenoids (Maroyi, 2023). Among the various secondary metabolites obtained from Fabaceae, prenylated (iso)flavonoids (i.e., prenylated isoflavonoids and flavonoids) are of particular interest. The addition of a prenyl group (a five-carbon isoprenoid substituent) to (iso)flavonoids enhances antimicrobial activity and the efflux pump-inhibitory activity against *S. aureus* (Kalli-Angel, 2021; Irianti et al., 2023). Prenyl substituents were found in some phytochemicals reported as NorA EPIs, such as nerol (Coelho et al., 2016), sophoraflavanone G (Sun et al., 2020), 5,7-dihydroxy-8-(2-methylbutanoyl)-6-[3,7-dimethylocta-2,6-dienyl]-4-phenyl-2H-chromen-2-one (Roy et al., 2013), 5,7-dihydroxy-6-(2-methyl-butanoyl)-8-(3-methylbut-2-enyl)-4-phenyl-2H-chromen-2-one (Roy et al., 2013), 5,7-dihydroxy-8-(2-methylbutanoyl)-6-(3-methylbut-2-enyl)-4-phenyl-2H-chromen-2-one (Roy et al., 2013), imperatorin (Joshi et al., 2014), and osthol (Joshi et al., 2014) (all the structures are shown in Supplementary Figure S1A). Among these reported phytochemicals with prenyl substituents, sophoraflavanone G is the only compound with a flavonoid backbone (Supplementary Figure S1A). Sophoraflavanone G showed a remarkable antibiotic potentiation effect, causing a 16-fold antibiotic MIC reduction at 2.4 µM (Sun et al., 2020). In addition to sophoraflavanone G, in our previous study revealed two other prenylated isoflavonoids, namely, neobavaisoflavone (isoflavone) and glabrene (isoflavene), as potential NorA EPIs in *S. aureus*, causing up to an 8-fold antibiotic MIC reduction in addition to their efflux inhibitory activity (Irianti et al., 2023). These findings further highlight the promising activity of prenylated (iso)flavonoids as potential NorA EPIs and their prospect to be developed further as potent NorA inhibitors.

Based on the characteristics of the top four EPI candidates (neobavaisoflavone, glabrene, glyceollin I, and glyceollin III) reported in our previous study, hydrophobicity and hydrophilic–lipophilic balance appear to play a crucial role in mediating the EPI activity (Irianti et al., 2023). Nevertheless, robust structure–activity relationships (SARs) of prenylated (iso)flavonoids as potential NorA EPIs remain to be established. To date, there have been limited quantitative structure–activity relationship (QSAR) studies of NorA EPIs involving phytochemical analogs. An example of a NorA EPI QSAR study using phytochemical analogs was performed by Nargotra et al., who used 25 analogs of piperine, which is a major constituent of *Piper nigrum* and *Piper longum* (Nargotra et al., 2009b). Piperine analogs potentiated ciprofloxacin by reducing the minimum inhibitory concentration (MIC) up to 8-fold in *norA-*overexpressing *S. aureus*, and the QSAR study highlighted the partial negative surface area as one of the key molecular features for NorA EPI activity (Nargotra et al., 2009b). Thus far, no QSAR model has been developed for prenylated (iso)flavonoids as potential NorA EPIs. Therefore, in this study, we aim to develop QSAR models for prenylated (iso)flavonoids as potential NorA inhibitors and propose new leading compounds for future research.

**FIGURE 1:**
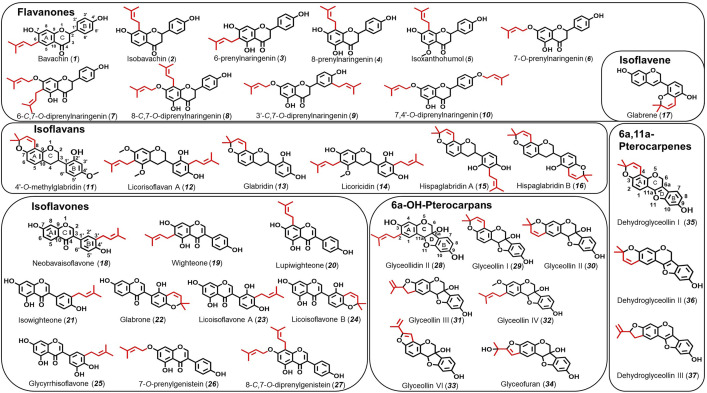
Overview of prenylated (iso)flavonoids from different subclasses evaluated in this study. Prenyl groups are highlighted in red, and A, B, C, and D rings in (iso)flavonoids are labeled.

In this research, we evaluated a total of 37 prenylated (iso)flavonoids, in contrast to our previous study, where we had a smaller dataset of only 11 compounds (Irianti et al., 2023). The activities of this extended collection of prenylated (iso)flavonoids from different subclasses (flavanones, isoflavans, isoflavene, isoflavones, pterocarpans, and pterocarpenes) with different numbers (mono- and di-prenylated) and different configuration of prenyl groups (ring, chain, and furan) were included (Figure 1). Then, the information obtained from the checkerboard and ethidium (Eth) accumulation assays was used to build binary classification QSAR models. A binary QSAR links the structures of the compounds through molecular descriptors with a “binary” expression of activity (i.e., 1 = active and 0 = inactive) (Gao et al., 1999). The developed binary QSAR models were used to systematically define and compare the important molecular features of prenylated (iso)flavonoids as potential NorA EPIs. Based on our previous work, we hypothesized that hydrophobicity, hydrophilic–lipophilic balance, and polar surface area are important molecular properties for prenylated (iso)flavonoids as potential NorA EPIs (Irianti et al., 2023). Moreover, because certain prenylated (iso)flavonoids exhibited both potential NorA EPI and antimicrobial activities (Irianti et al., 2023), we compared the key molecular properties associated with potential NorA EPI activity (identified in this study) to those linked to antimicrobial activity against MRSA, as determined by a previous QSAR model (Kalli et al., 2021). This comparison allowed us to identify which distinct properties defined prenylated (iso)flavonoids as potential NorA EPIs or as antimicrobials. Last, the 3D optimized structures of the candidate prenylated (iso)flavonoids were aligned with that of the known NorA EPI PQQ16P (Felicetti et al., 2018; Palazzotti et al., 2023) to highlight common pharmacophoric features and to propose possible structural improvements for prenylated (iso)flavonoids as potential NorA EPIs.

## 2 Materials and methods

### 2.1 Materials

Fluoroquinolone-resistant and *norA-*overexpressing strain of *S*. *aureus* (SA)-1199B and *norA-*knockout strain (SA-K1758) were obtained from BEI resources, as previously described (Irianti et al., 2023). Ethidium bromide (EtBr), propidium iodide (PI), phosphate buffer saline (PBS) (pH 7.4), reserpine, ciprofloxacin, and erythromycin were obtained from Sigma-Aldrich (United States). Dimethyl sulfoxide (DMSO) was purchased from Brunschwig Chemie B.V. (Netherlands), and ethyl acetate absolute was purchased from Biosolve B.V. (Netherlands). Peptone physiological salt solution (PPS) was obtained from Tritium Microbiologie (Netherlands). Tryptone soy agar (TSA) and tryptone soy broth (TSB) were purchased from Oxoid Ltd. (United Kingdom). Transparent 96-well plates (655161) and black 96-well plates (655900) were purchased from Greiner Bio-One transparent (Austria).

Prenylated isoflavonoids, namely, bavachin (***1***), isobavachin (***2***), 6-prenylnaringenin (***3***), isoxanthohumol (***5***), wighteone (***19***), lupiwighteone (***20***), isowighteone (***21***), licoisoflavone A (***23***), and glycyrrhisoflavone (***25***), were purchased from ChemFaces (China). 8-Prenylnaringenin (***4***), 7-*O*-prenylnaringenin (***6***), 6-*C*,7-*O*-diprenylnaringenin (***7***), 8-*C*,7-*O*-diprenylnaringenin (***8***), 3′-*C*,7-*O*-diprenylnaringenin (***9***), 7,4′-*O*-diprenylnaringenin (***10***), 7-*O*-prenylgenistein (***26***), and 8-*C*,7-*O*-diprenylgenistein (***27***) were previously synthesized and characterized (Ritsema et al., 2025). Glabridin was purchased from Wako (Japan). Neobavaisoflavone (***18***) was purchased from PhytoLab GmbH & Co. KG (Germany). Glabrene (***17***) was purchased from Arctom (United States). Licorisoflavan A (***12***), licoricidin (***14***), hispaglabridin A (***15***), glabrone (***22***), and licoisoflavone B (***24***) were previously purified from *Glycyrrhiza* spp. roots and characterized (van Dinteren et al., 2025). 4′-*O*-Methylglabridin (***11***) and hispaglabridin B (***16***) were previously purified from *Glycyrrhiza* spp. roots and characterized (Van de Schans et al., 2015). Pterocarpan and pterocarpene compounds, namely, glyceollidin II (***28***), glyceollin I (***29***), glyceollin II (***30***), glyceollin III (***31***), glyceollin IV (***32***), glyceollin VI (***33***), glyceofuran (***34***), dehydroglyceollin I (***35***), dehydroglyceollin II (***36***), and dehydroglyceollin III (***37***) were previously purified from soybeans (*Glycine max* (L.) Merrill) and characterized (Van De Schans et al., 2016). The identity and purity of all prenylated (iso)flavonoids were confirmed by reversed-phase ultrahigh-pressure liquid chromatography coupled to photo diode array and ion trap mass spectrometry detection (RP-UHPLC-PDA-IT-MS^n^), and proton nuclear magnetic resonance spectroscopy (^1^H NMR) was additionally performed for the synthesized or purified compounds (not the purchased ones). Prenylated (iso)flavonoids showed UV purities ≥80%, except licoricidin, glabrone, and glycyrrhisoflavone. The details of the compounds’ purities are provided in Supplementary Table S1.

### 2.2 Antibiotic potentiation (checkerboard) assay

The checkerboard assays were conducted with SA-1199B (with all 37 prenylated (iso)flavonoids) and SA-K1758 (only with the best candidate compounds, namely, glabrene, neobavaisoflavone, 8-prenylnaringenin, wighteone, licoisoflavone A, and glycyrrhisoflavone), as described previously with slight modifications (Irianti et al., 2025). One colony was grown in 3 mL TSB and further incubated overnight at 37 °C and 180 rpm. Stock solutions of phytochemicals (prepared in DMSO), along with ciprofloxacin or erythromycin (prepared in PBS and 0.1% v/v acetic acid), were diluted in TSB. Both phytochemicals and antibiotic (ciprofloxacin or erythromycin) TSB solutions were prepared at four times the final test concentrations. Phytochemicals were tested at a final concentration of 10, 20, and 40 µM. An overnight culture of bacteria was diluted in TSB to an OD_600_ of 0.012 (final OD_600_ of 0.006 in a 96-well plate). Next, 50 µL of each compound (phytochemical and antibiotic) was combined with 100 µL of the diluted culture in a 96-well plate, resulting in a final bacterial inoculum of 6.92 ± 0.29 log10 CFU/mL. Column 1 consisted of a serial dilution of antibiotic only, and row H consisted of a serial dilution of phytochemicals alone, which were subsequently used to determine the minimum inhibitory concentration (MIC) for each antibiotic and phytochemical. The 96-well plates (Greiner Bio-One transparent, Austria) were incubated at 200 rpm at 37 °C in a shaker incubator (Innova 42R, Eppendorf, Germany). Bacterial growth (OD_600_) was measured using a Tecan Infinite 200 Pro M nanoplate reader before and after 20 h of incubation. Experiments were conducted in three biological replicates. Final OD values were determined by subtracting the initial absorbance (ΔOD_600_). The concentration of compounds where samples showed ΔOD_600_ ≤ 0.18 was considered as the minimum inhibitory concentration (MIC). The fold-reduction (FR) was calculated by comparing the MIC of the antibiotic alone relative to the lowest MIC of the antibiotic in combination with phytochemicals (Equation 1).

$$\text{Fold reduction }\left(\text{FR}\right)=\frac{{MIC}_{antibiotic}}{{MIC}_{antibiotic+EPI}}.$$ (1)

### 2.3 Eth accumulation assay

Eth accumulation assays were performed according to previous work (Irianti et al., 2023). Due to the limited availability of phytochemicals, Eth accumulation assay of all the prenylated (iso)flavonoids collection was performed only with SA-1199B. The OD_600_ of the overnight culture was adjusted to 0.6–0.8, with an average inoculum of 9.01 ± 0.13 log_10_ CFU/mL. Fold changes (FC) in increased Eth accumulation were calculated by comparing the final fluorescence unit (RFU) relative to the negative control at t = 60 min and presented as the means with a standard deviation (Irianti et al., 2023). All samples were measured in three biological replicates in technical triplicates.

### 2.4 Membrane permeability assay

Propidium iodide (PI) was used to evaluate the membrane permeabilization effect of all 37 prenylated (iso)flavonoids in SA-1199B, as previously reported with slight modifications (Irianti et al., 2023). After the washing step, the OD_600_ was adjusted to 0.6–0.8 with an average inoculum size of 9.18 ± 0.32 log10 CFU/mL. Experiments were performed in three biological replicates in technical triplicates. The permeabilization activity was represented as a percentage of propidium uptake and calculated with Equation 2.

$$\text{Propidium uptake }\left(\%\right)=\frac{\mathrm{Exp}.\;value-low\;control}{high\;control-low\;control}\times 100.$$ (2)

The *experimental (Exp.) value* represents the observed relative propidium uptake, which was calculated by taking the final fluorescence unit (RFU) at t = 60 min relative to the negative control. *Low control* denotes the relative propidium uptake in untreated cells (negative control), and *high control* denotes the relative propidium uptake in permeabilized cells, which were prepared by heating the cells for 10 min at 95 °C (positive control).

### 2.5 EPI definitions used in this study

Despite extensive research on EPI discovery, there is no consensus on the definition used to categorize EPI and non-EPI compounds. Therefore, in this study, we developed a classification method based on two assays to define and propose leading EPI candidates. EPI and non-EPI compounds were classified based on the maximum fold reduction in ciprofloxacin MIC (FR_CIP_) from three biological repetitions of the checkerboard assays and the fold change in increased ethidium accumulation (FC) obtained from Eth accumulation assays using SA-1199B. Due to the antimicrobial properties of most prenylated (iso)flavonoids (Kalli et al., 2021; Long et al., 2022), 10 µM was the chosen concentration to compare the EPI activity via checkerboard and Eth accumulation assays as this is a sub-inhibitory concentration for all the tested compounds. The threshold of FR_CIP_ > 2 is considered a notable antibiotic potentiation effect, as a 2-fold reduction (FR_CIP_ = 2) could still take place due to biological variation. Meanwhile, the threshold of FC > 1.2 was considered a notable Eth accumulation by comparing with the FC in Eth accumulation of the reported NorA EPI reserpine. The summary of EPI definitions used in this study is shown in Table 1. “EPIs” were defined as compounds that showed notable FR_CIP_ (FR_CIP_ > 2) and FC (FC > 1.2) at a concentration of 10 μM, which was equal to or lower than ¼ MIC (10 µM ≤ ¼ MIC). Antimicrobial EPIs were defined as compounds with notable FR_CIP_ and FC at a concentration of 10 μM, which was equal to or higher than ½ MIC, and were labeled as “EPI (antimicrobial).” This distinction was made because the notable FR_CIP_ and FC observed at or above ½ MIC might be affected by the antimicrobial properties of the compounds. Compounds that did not meet the FR_CIP_ and/or FC thresholds at 10 µM were categorized as non-EPI.

**TABLE 1:** Overview of the EPI and non-EPI categories used in this study.

FC > 1.2	FR _CIP_ > 2	Tested concentration	Category
Yes	Yes	10 µM ≤ ¼ MIC	EPI
Yes	Yes	10 µM ≥ ½ MIC	EPI with antimicrobial (EPI-Ant)
Yes	No	10 µM	Non-EPI
No	Yes	10 µM	Non-EPI
No	No	10 µM	Non-EPI

FC and FR_CIP_ are defined as fold-change in Eth accumulation and the maximum fold reduction in ciprofloxacin MIC, respectively

### 2.6 Binary classification QSAR model

Binary classification models were developed according to previous work by Kalli et al. with some modifications (Kalli et al., 2022). Binary QSAR models for EPIs were built using FR_CIP_ and the FC as response variables using the threshold values described in Section 2.5. In general, SMILES codes of the 37 prenylated (iso)flavonoids were obtained from PubChem or, when not available, the chemical structures were drawn manually using ChemDraw (PerkinElmer, version 18.1.2.18), and the SMILES codes were retrieved and imported to Molecular Operating Environment (MOE, v.2019.08, Chemical Computing Group). Partial charge correction followed by energy minimization was performed using MMFF94x as a force field and a root mean square (RMS) gradient of 0.01 kcal/mol/A^2^. Afterward, a conformational search was conducted using lowModeMD (RMS gradient 0.1 kcal/mol/A^2^, rejection limit = 100, conformation limit = 1, and iteration limit = 10,000 as in default setting). The best conformer was then used to calculate molecular descriptors (2D and i3D) in MOE. Subsequently, the molecular database was curated from descriptors that (i) had constant values or less than five different values (no variability), (ii) were highly intercorrelated (R_pearson_ > 0.99) (multicollinearity), and (iii) were complex in interpretation. As a result, 163 descriptors were present in the final dataset (the complete list of selected descriptors is provided in Supplementary Table S2).

Because of the relatively small dataset (37 molecules), binary QSAR models were constructed using the whole dataset, without splitting, using either FR_CIP_ or FC as independent variables. The binary QSAR uses statistical probability estimation to predict the active and inactive compounds (Labute et al., 2002). The performance and validation of binary QSAR models was evaluated by assessing the total accuracy (A), accuracy on actives (A1), accuracy on inactives (A0), and the cross-validated accuracies [total (XA), active (XA1), and inactive (XA0)] (Kalli et al., 2022). The cross-validation of the binary QSAR model was obtained using a leave-one-out procedure (Gao et al., 1999). The binary QSAR models from 2–5 descriptors were built and evaluated for their accuracy. To check the multicollinearity of the selected models, variance inflation factors (VIFs) analysis was performed. The VIF value is calculated as 1/(1-*r* ^*2*^), where *r* ^*2*^ is the squared correlation coefficient between two molecular descriptors. A VIF<5 indicates the absence of collinearity (Nargotra et al., 2009a).

Within MOE, component limit and smoothing were optimized and adjusted to obtain the best binary QSAR model (Gao et al., 1999). The component limit specifies the number of principal components to be used in the model, and the smoothing parameter, also called observation error, is used to minimize the sensitivity of the derived model (Gao et al., 1999; Labute et al., 2002). To obtain the best model (highest accuracy), a component limit between 2 and 5 and a smooth factor between 0.08 and 0.25 were explored in generating the QSAR models, as previously described (Gao et al., 1999). The best model with the FR_CIP_ dataset was obtained by using a component limit of three and a smooth factor of 0.15 or 0.16. Meanwhile, a component limit of four and a smooth factor of 0.13 generated the best QSAR model with the FC dataset. All the binary QSAR performances are shown in Supplementary Table S3.

### 2.7 Comparison of EPI with and without antimicrobial activity

Molecular descriptors were calculated as previously described in Section 2.6. Then, the key molecular descriptors defining good EPI activity based on our developed QSAR model were compared with the key molecular descriptors defining good antimicrobial activity against MRSA, obtained from a previous QSAR study for prenylated (iso)flavonoids (Kalli et al., 2021). For each molecular descriptor, the statistical difference between the mean values of the two groups (EPIs with antimicrobial activity and EPIs without antimicrobial activity) was calculated with GraphPad Prism 9 using a Student’s t-test with Welch’s correction (Ahad and Yahaya, 2014).

### 2.8 Structural alignment of prenylated (iso)flavonoids with PQQ16P

The structures of prenylated (iso)flavonoids were aligned with a 2-phenylquinolone derivative, namely, PQQ16P, a synthetic NorA EPI (Felicetti et al., 2018; Palazzotti et al., 2023), which has structural similarities with prenylated (iso)flavonoids. The alignment of prenylated (iso)flavonoids with PQQ16P was performed in MOE (v.2019.08, Chemical Computing Group). The compounds were energy-minimized as previously described in Section 2.6 and subsequently aligned with a flexible mode iteration limit of 100 and a failure limit of 10. The alignment was performed in three repetitions and evaluated based on the alignment score (S value), with lower values indicating better alignments.

### 2.9 Pharmacophore elucidation

A pharmacophore model was generated using the pharmacophore elucidation query module of MOE (active coverage 1.0, query spacing 0.6, feature limit 5, and query cluster 1.25). PQQ16P and the best-aligned prenylated (iso)flavonoids (S value < −80) were energy-minimized and used to extract common pharmacophoric features. The best pharmacophore model was selected based on the overlap score and the observed molecular features.

## 3 Results and discussion

### 3.1 Potential NorA EPI activity of prenylated (iso)flavonoids

**FIGURE 2:**
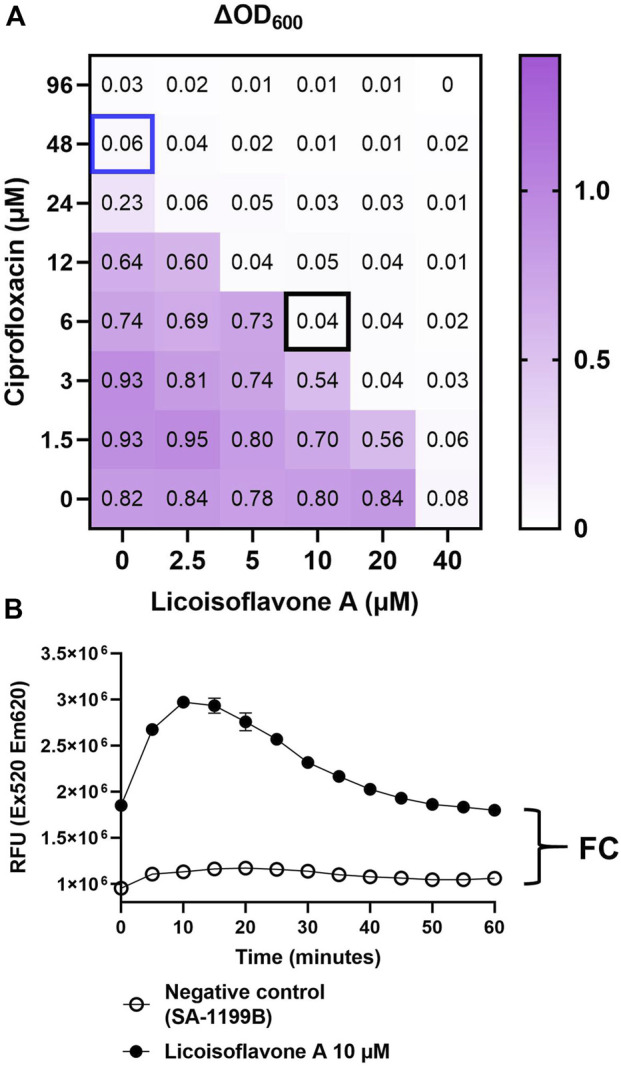
The representatives of **(A)** checkerboard results of licoisoflavone A in combination with ciprofloxacin, and **(B)** Eth accumulation result of licoisoflavone A. Both experiments were performed in a *norA-*overexpressing strain (SA-1199B).

The potential NorA EPI activities of 37 prenylated isoflavonoids were evaluated in *norA-*overexpressing *S. aureus* based on the antibiotic potentiation effect of the compounds and Eth accumulation assays (Irianti et al., 2023). In this study, the antibiotic (ciprofloxacin) potentiation effect (FR_CIP_) of prenylated (iso)flavonoids was determined as the ratio between the MIC of ciprofloxacin alone and its MIC in combination with prenylated (iso)flavonoids at 10 µM. Figure 2A shows the results of checkerboard assay of licoisoflavone A. The MIC of ciprofloxacin alone (48 μM, blue box) was divided by the MIC of ciprofloxacin in combination with licoisoflavone A (***23***) at 10 µM (6 μM, black box), which resulted in a fold-reduction of 8 (Figure 2A). In addition to that, efflux inhibition is also needed to select active EPIs, which is determined via FC increase in Eth accumulation after 1 h of measurement (Figure 2B). The FR_CIP_ and FC values of all 37 prenylated (iso)flavonoids are described in Table 2.

**TABLE 2:** Summary of the EPI activities of 37 prenylated isoflavonoids from different subclasses against *norA-*overexpressing *S. aureus* (SA-1199B)^a^.

Subclass	Name	MIC compounds alone (µM)	At 10 µM phytochemical		EPI/non-EPI at 10 µM
			Cip MIC fold reduction (FR_CIP_)	Average fold-change Eth accumulation (FC) ± St. dev	
Flavanones					
(***1***)	Bavachin	>40	4	1.1 ± 0.1	Non-EPI^a^
(***2***)	Isobavachin	>40	2–4	0.9 ± 0.1	Non-EPI
(***3***)	6-Prenylnaringenin	40	4–8	1.0 ± 0.1	Non-EPI
(***4***)	8-Prenylnaringenin	40	4–8	1.3 ± 0.1	EPI
(***5***)	Isoxanthohumol	>40	2	1.0 ± 0.1	Non-EPI
(***6***)	7-*O*-Prenylnaringenin	20	2–4	2.0 ± 0.2	EPI-Ant
*(* ***7*** *)*	6-*C*,7-*O*-Diprenylnaringenin	>40	2–4	1.5 ± 0.2	EPI
(***8***)	8-*C*,7-*O*-Diprenylnaringenin	>40	1–2	1.6 ± 0.4	Non-EPI
(***9***)	3′-*C*,7-*O*-Diprenylnaringenin	>40	1–2	1.8 ± 0.3	Non-EPI
(***10***)	7,4′-*O*-Diprenylnaringenin	>40	1–2	1.0 ± 0.1	Non-EPI
Isoflavans					
(***11***)	4′-*O*-Methylglabridin	20	4–8	1.5 ± 0.2	EPI-Ant
(***12***)	Licorisoflavan A	>40	1–2	1.4 ± 0.2	Non-EPI
(***13***)	Glabridin	20–40	2–4	2.4 ± 0.4	EPI-Ant
(***14***)	Licoricidin	20	8–16	1.5 ± 0.2	EPI-Ant
(***15***)	Hispaglabridin A	20–40	4	1.4 ± 0.2	EPI-Ant
(***16***)	Hispaglabridin B	≥40	2	2.4 ± 0.3	Non-EPI
Isoflavene					
(***17***)	Glabrene	>40	4	1.3 ± 0.2	EPI
Isoflavones					
(***18***)	Neobavaisoflavone	40	4	1.4 ± 0.2	EPI
(***19***)	Wighteone	40	4–8	1.3 ± 0.1	EPI
(***20***)	Lupiwighteone	>40	2	1.0 ± 0.1	Non-EPI
(***21***)	Isowighteone	>40	2	0.9 ± 0.1	Non-EPI
(***22***)	Glabrone	40	4–8	1.0 ± 0.1	Non-EPI
(***23***)	Licoisoflavone A	40	8	1.6 ± 0.1	EPI
(***24***)	Licoisoflavone B	20	8–16	1.5 ± 0.3	EPI-Ant
(***25***)	Glycyrrhisoflavone	>40	2–4	1.3 ± 0.1	EPI
(***26***)	7-*O*-Prenylgenistein	>40	1–2	1.0 ± 0.1	Non-EPI
(***27***)	8-*C*,7-*O*-Diprenylgenistein	>40	1–2	1.0 ± 0.0	Non-EPI
6a-OH-Pterocarpan					
(***28***)	Glyceollidin II	>40	1–2	1.0 ± 0.1	Non-EPI
(***29***)	Glyceollin I	>40	1–2	1.0 ± 0.1	Non-EPI
(***30***)	Glyceollin II	>40	1–2	1.0 ± 0.1	Non-EPI
(***31***)	Glyceollin III	>40	2	0.9 ± 0.1	Non-EPI
(***32***)	Glyceollin IV	>40	1–2	1.1 ± 0.1	Non-EPI
(***33***)	Glyceollin VI	>40	2–4	1.0 ± 0.1	Non-EPI
(***34***)	Glyceofuran	>40	1	1.0 ± 0.1	Non-EPI
6a,11a-Pterocarpene					
(***35***)	Dehydroglyceollin I	20	2–4	1.4 ± 0.2	EPI-Ant
(***36***)	Dehydroglyceollin II	20	2–4	1.2 ± 0.1	Non-EPI
(***37***)	Dehydroglyceollin III	>40	2–4	1.1 ± 0.1	Non-EPI
Others	Reserpine	>40	2–4	1.3 ± 0.1	EPI
	Negative control	-	-	1.0 ± 0.1	​

^a^ The MIC of ciprofloxacin alone was 24 μM–48 μM, as described in Supplementary Table S4. The criteria for EPI and non-EPI were previously described in Table 1.

The antimicrobial activities and membrane permeabilizing effects of prenylated (iso)flavonoids (Table 2; Supplementary Figure S2) were checked to avoid false-positive results, as some prenylated (iso)flavonoids are good antimicrobials and membrane permeabilizers (Araya-Cloutier et al., 2018; Kalli et al., 2021).

Based on the defined EPI criteria (as described in Section 2.5) (Table 1), in addition to our previously reported potential NorA EPIs glabrene (***17***) and neobavaisoflavone (***18***) (Irianti et al., 2023), five new prenylated (iso)flavonoid compounds, namely, 8-prenylnaringenin (***4***), 6-*C*,7-*O*-diperenylnaringenin (***7***), wighteone (***19***), licoisoflavone A (***23***), and glycyrrhisoflavone (***25***), exhibited efflux inhibition and potentiated ciprofloxacin at 10 µM without having antimicrobial activity. Compared to our previous study (Irianti et al., 2023), the FR_CIP_ of glabrene in this study was two times higher (2-fold in the previous study vs. 4-fold in this study). This 2-fold difference in the FR_CIP_ might be affected by biological variance or different experimental settings. Among these five new compounds, 8-prenylnaringenin (***4***), wighteone (***19***), and licoisoflavone A (***23***) exhibited better antibiotic potentiation activity (up to 8-fold) than glabrene (***17***) and neobavaisoflavone (***18***) at ¼ MIC. Moreover, these three compounds exhibited a better potentiation effect than reserpine (up to 4-fold) at the same concentration. Compared to sophoraflavanone G, a *C*10-prenylated flavonoid (Supplementary Figure S1A), the top three prenylated (iso)flavonoids demonstrated less antimicrobial activity (MIC of 40 µM). Although sophoraflavanone G potentiated fluoroquinolone antibiotic norfloxacin up to 16-fold at four times lower concentration than prenylated (iso)flavonoids, it showed very good antimicrobial activity (MIC of 9.4 µM) (Sun et al., 2020). The lower antimicrobial properties of our top three prenylated (iso)flavonoids make them interesting for further studies as potential NorA EPIs, as antimicrobial properties are less desired for EPI development (Sharma et al., 2019).

None of the compounds from the pterocarpan and pterocarpene subclasses exhibited promising efflux inhibition and antibiotic potentiation activities in *norA-*overexpressing *S. aureus* at 10 µM. In our previous work, we demonstrated that pterocarpans glyceollin I (***29***) and glyceollin III (***31***) showed notable activity as potential NorA EPIs, but only at ten times higher concentrations than the concentration used in the current study (Irianti et al., 2023). It should be taken into account that the classification of EPI and non-EPI (shown in Table 2) was based on the activity observed (FR_CIP_ and FC) at the set threshold of 10 µM (as explained in Section 2.5; Table 1). Nonetheless, some “non-EPI” compounds could potentially still be active as potential EPIs at higher concentrations.

### 3.2 Specificity of potential NorA EPI candidates

**FIGURE 3:**
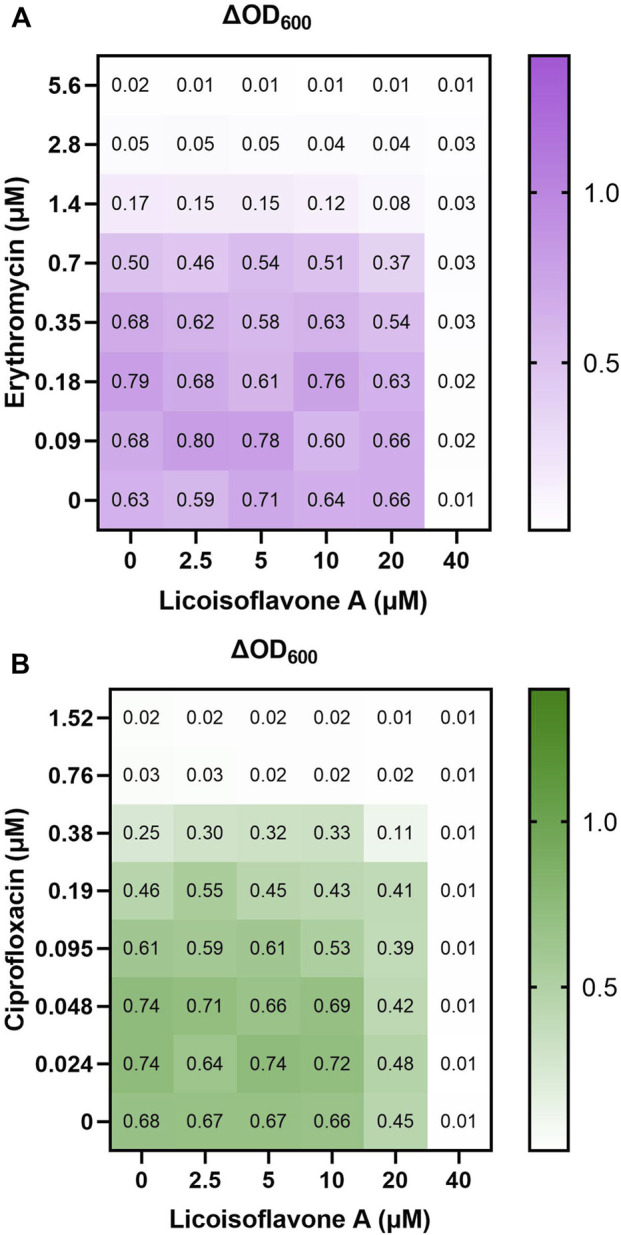
Minimal antibiotic potentiation effect (represented by ΔOD_600_) shown in the checkerboard results of licoisoflavone A in combination with **(A)** erythromycin in a *norA-*overexpressing strain and **(B)** ciprofloxacin in a *norA-*knockout strain. The numbers represent the ΔOD_600_ before and after 20 h of growth. The color code indicates no growth (white) up to strong growth (dark color).

In addition to remarkable ciprofloxacin potentiation and an increased level of Eth accumulation in the *norA-*overexpressing strain, a NorA EPI should neither show remarkable antibiotic potentiation with a primarily non-NorA substrate (such as erythromycin) nor in a *norA-*knockout strain (FR_CIP_ ≤ 2), as shown in Figures 3A,B with the representative prenylated compound licoisoflavone A (***23***). The other top EPI candidates from the first screening were tested similarly, except compound 6-*C*,7-*O*-diprenylnaringenin (***7***), due to its limited availability.

As shown in Table 3, all tested compounds showed minimal antibiotic potentiation effect (FR_ERY_ 1–2) in the presence of erythromycin, which is not the main substrate of NorA (Reynolds et al., 2003; Poole, 2007). Moreover, no remarkable potentiation with these prenylated isoflavonoids was seen in the *norA-*knockout strain in the presence of ciprofloxacin (FR_CIP_ 1–2), which further supports their activity against NorA.

**TABLE 3:** Checkerboard results of the most promising EPI candidates in the presence of erythromycin (ERY) and ciprofloxacin (CIP) in SA-1199B and SA-K1758, respectively.

Compound	SA-1199B (*norA*+++)		SA-K1758 (Δ*norA*)	
	MIC of compound alone (µM)	FR of MIC_ERY_ in combination (FR_ERY_)	MIC of compound alone (µM)	FR of MIC_CIP_ in combination (FR_CIP_)
Antibiotic (AB)	1.4–2.8*	n.a.^a^	0.38–0.76**	n.a.^a^
+8-Prenylnaringenin (***4***)	40	2	40	2
+ Glabrene (***17***)	>40	1–2	40	2
+ Neobavaisoflavone (***18***)	40	1–2	20	2
+ Wighteone (***19***)	40	2	20	1–2
+ Licoisoflavone A (***23***)	40	1–2	40	2
+ Glycyrrhisoflavone (***25***)	40	1	40	2

*MIC of antibiotic erythromycin.

**MIC of antibiotic ciprofloxacin.

^a^ N.a., not applicable.

The possibility that these prenylated (iso)flavonoids inhibit other pumps in *S. aureus* cannot be completely excluded, as minor Eth accumulation in the *norA-*knockout strain K-1758 was observed in our previous study (Irianti et al., 2023). Therefore, to validate the specificity of prenylated (iso)flavonoids for NorA, future research should involve the use of a “clean” complemented NorA-upregulated mutant, as previously suggested (Laws et al., 2021).

### 3.3 Effect of structural changes in prenylated (iso)flavonoids on their potential NorA EPI activity

**FIGURE 4:**
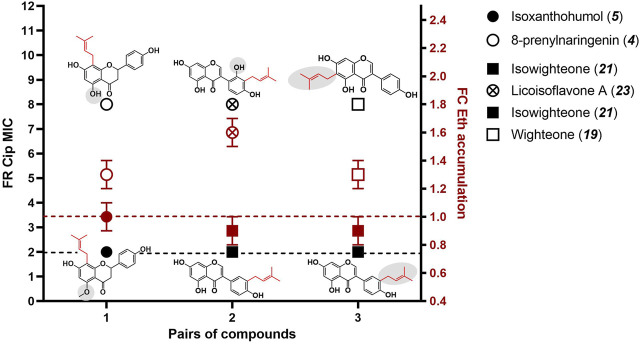
Structure–activity relationship (SAR) analysis of the top-three potential EPIs in comparison with non-EPI analogs. The structural differences between EPI and non-EPI analogs are shown in gray. The dashed lines indicate the minimum of antibiotic potentiation effect (FR_CIP_) of 2-fold (black) and the minimum Eth accumulation (FC) of 1-fold (red).

To analyze the effect of structural changes on the potential NorA EPI activity of prenylated (iso)flavonoids, SARs were explored using some prenylated (iso)flavonoids in this study. The SARs were established by analyzing structural differences between the top three compounds, which showed FR_CIP_ up to 8-fold, in comparison with their inactive EPI analogs (at 10 µM), which are further referred as non-EPI. The three pairs of EPI and non-EPI analogs demonstrated that the number of -OH groups and prenyl locations influenced the EPI activity of prenylated flavanones and isoflavones (Figure 4; Supplementary Figure S3). Based on SARs (Figure 4), some main conclusions can be derived:

1. The presence of -OH groups influenced the potential NorA EPI activity of prenylated flavanones and isoflavones. In some cases, hydroxylation increased the NorA EPI activity: from isoxanthohumol (***5***, non-EPI) to 8-prenylnaringenin (***4***, EPI) and from isowighteone (***21***, non-EPI) to licoisoflavone A (***23***, EPI) and to glycyrrhisoflavone (***25***, EPI) (Figure 4; Supplementary Figure S3). In other cases, the removal of an -OH group resulted in an increased EPI activity, as observed from isowighteone (***21***, non-EPI) to neobavaisoflavone (***18***, EPI) (Supplementary Figure S3).
2. The position of prenylation was also associated with the potential NorA EPI activities in prenylated flavanones and isoflavones. As shown in Figure 4, *C*6 prenylation improved the NorA EPI activities of isoflavones, as observed in isowighteone (***21***, non-EPI) vs. wighteone (***19***, EPI). The same trend was also observed for lupiwighteone (***20***, non-EPI) vs. wighteone (***19***, EPI) (Supplementary Figure S3). In contrast, *C*8 prenylation was favorable for EPI activity in flavanones, as observed in 8-prenylnaringenin (***4***, EPI) vs. 6-prenylnaringenin (***3***, non-EPI) (Supplementary Figure S3). The effect of prenyl location on EPI activity appears to be comparable to the effect of prenyl location observed in antibacterial activity against *S. aureus* (Sato et al., 2006; Zhou and Wan, 2015).

The role of -OH groups in the efflux inhibition of (unprenylated) flavonoids in SA-1199B has been previously reported, wherein the additional -OH group in epigallocatechin gallate resulted in decreased efflux inhibition relative to epicatechin gallate (Gibbons et al., 2004; Waditzer and Bucar, 2021). Moreover, the role of prenyl configuration was reported as one of the key molecular properties involved in the antimicrobial activity of prenylated (iso)flavonoids (Araya-Cloutier et al., 2018). Within our dataset of flavanone and isoflavone compounds, changes in prenyl configuration appeared to affect the potential NorA EPI activity, but no clear SAR could be proposed (Supplementary Figure S3). Overall, our findings highlighted that primarily, the number of -OH groups and the prenyl position are linked to the potential EPI activity of prenylated (iso)flavonoids from the flavanone and isoflavone subclasses.

### 3.4 (Quantitative) structure–activity relationship and molecular properties of prenylated (iso)flavonoids as potential NorA EPIs

Using the EPI activities (checkerboard and Eth accumulation assays) and molecular structures of the collection of prenylated (iso)flavonoids tested (Table 2), robust binary classification QSAR models were built to obtain overall insight into the molecular features underlying potential NorA EPI activity by using the maximum FR and FC as dependent variables. The best model was chosen based on all accuracies (A, A1, A0, XA, XA1, and XA0) that were higher than 75%. Two descriptor models were chosen due to their balanced statistical performance, with total accuracy and cross-validated accuracy ≥75% (Supplementary Figures S4A,B), as well as to avoid overfitting (Kalli et al., 2022).

#### 3.4.1 QSAR binary model from the FR dataset

The binary classification QSAR model from the FR_CIP_ dataset was generated to know the molecular properties of prenylated (iso)flavonoids in potentiating ciprofloxacin (Supplementary Table S3A). Table 4 summarizes the best binary classification model of two molecular descriptors, *PEOE_VSA_FPPOS* and *vsurf*_HL1, that explained the ciprofloxacin potentiation activity of prenylated isoflavonoids. This best model obtained with the FR dataset had a total accuracy of 89% and a cross-validated accuracy of 78%. The first descriptor *PEOE_VSA_FPPOS* refers to the fractional positive polar van der Waals surface area. It is calculated as the sum of the surface area (*v*_i_) for which the partial charge (*q*_i_) is greater than 0.2, divided by the total surface area (Molecular Operating Environment (MOE), 2024). The second descriptor is *vsurf_HL1*, which represents the hydrophilic–lipophilic balance (Molecular Operating Environment (MOE), 2024). The *vsurf_*HL1 can be linked to the interaction of molecules with the hydrophilic and hydrophobic part of the biological target through their surface properties, such as hydrophobicity, hydrogen bond, shape, and electrostatic properties (Moorthy et al., 2014). The binary FR_CIP_ QSAR model with *PEOE_VSA_FPPOS* and *vsurf_HL1* descriptors indicated that both the fractional positive polar van der Waals surface area and balanced hydrophilicity and hydrophobicity influence their antibiotic’s potentiating activities. Within 37 prenylated (iso)flavonoids, it was observed that potential NorA EPIs (without antimicrobial activity) showed *PEOE_VSA_FPPOS* in a range of 0.05–0.16, and their *vsurf_*HL1 values were in a range of 0.03–0.14 (Supplementary Figure S5A; Supplementary Table S5).

**TABLE 4:** Accuracy and cross-validated accuracy of the best binary QSAR model developed with the FR_CIP_ dataset in this study.

Total descriptors	Descriptor importance	Descriptors	VIF	Accuracy			Cross-validated accuracy		
				Total (A)	Actives (A1)	Inactives (A0)	Total (XA)	Actives (XA1)	Inactives (XA0)
2	0.38 0.36	*PEOE_VSA_FPPOS* *vsurf_*HL1	2.94	89%	90%	88%	78%	81%	75%

#### 3.4.2 QSAR binary model from the FC dataset

To gain insight into the molecular properties associated with Eth accumulation, the binary QSAR model was built from the FC dataset. Similar to the QSAR binary from the FR_CIP_ dataset, the best binary classification model from the FC dataset was selected based on its overall performance accuracy (Supplementary Table S3B). Table 5 describes the best binary classification model obtained with two molecular descriptors, *log*P(o/w) and *PEOE_VSA_FPNEG*, that explained the FC in Eth accumulation. The best model obtained from the FC dataset had total and cross-validated accuracies of 84% and 78%, respectively. Descriptor *log*P(o/w) refers to the log of the octanol/water partition coefficient (Molecular Operating Environment (MOE), 2024), which is related to hydrophobicity. Descriptor *PEOE_VSA_FPNEG* represents the fractional negative polar van der Waals surface area, which is the sum of the *v*_i_ such that the *q*_i_ is less than −0.2, divided by the total surface area (Molecular Operating Environment (MOE), 2024). The active EPIs (without antimicrobial activity) showed *log*P(o/w) between 2 and 5 and *PEOE_VSA_FPNEG* in a range of 0.07–0.14 (Supplementary Figure S5B; Supplementary Table S5).

**TABLE 5:** Accuracy and cross-validated accuracy of the best binary QSAR model developed with the FC dataset in this study.

Total descriptors	Descriptor importance	Descriptors	VIF	Accuracy			Cross-validated accuracy		
				Total (A)	Actives (A1)	Inactives (A0)	Total (XA)	Actives (XA1)	Inactives (XA0)
2	0.45 0.33	*log*P(o/w) PEOE_VSA_FPNEG	3.47	84%	83%	84%	78%	78%	79%

VIF was used to check the multicollinearity of two descriptors in the model. VIF<5 is defined as low multicollinearity.

It is interesting to highlight that descriptors *PEOE_VSA_FPPOS* (Table 4) and *PEOE_VSA_FPNEG* (Table 5) are positively correlated (R_pearson_ = 0.87), which indicates that these descriptors explain the same variation in the data. Overall, the obtained binary classification models can explain the FR_CIP_ and FC variation in the datasets with high levels of accuracy. Moreover, the selected descriptors highlight that the fractional polar van der Waals surface area, hydrophilicity–hydrophobicity balance, and overall hydrophobicity of prenylated (iso)flavonoids play a role in efflux inhibition and antibiotic potentiation activity. The importance of hydrophobicity and hydrophilic–lipophilic balance is consistent with our previous study (Irianti et al., 2023) with a smaller subset of prenylated (iso)flavonoids. Moreover, the role of the fractional polar van der Waals surface area observed in this study was in line with the result of the QSAR study with piperine analogs (Nargotra et al., 2009b), where the partial charge surface area is involved in NorA EPI activity. Yet, external validation of the developed QSAR models with a different set of prenylated (iso)flavonoid compounds should be performed to completely validate the proposed binary models.

#### 3.4.3 Distinct molecular properties of prenylated (iso)flavonoids as potential NorA EPIs and as antimicrobials against *S. aureus*

Previous studies have demonstrated antimicrobial properties of prenylated (iso)flavonoids against *S. aureus* and MRSA (Kalli et al., 2021; Long et al., 2022). Furthermore, the interplay between antimicrobial and potential NorA EPI activity of some prenylated (iso)flavonoids was already observed in our previous study (Irianti et al., 2023) and in this current study. It was found that some prenylated (iso)flavonoids were active as EPIs and had antimicrobial activity, such as 7-*O*-prenylnaringenin (***6***), 4′-*O*-methylglabridin (***11***), glabridin (***13***), licoricidin (***14***), hispaglabridin A (***15*** *)*, licoisoflavone B (***24***), and dehydroglyceollin I (***35***) (Table 2). To explore if there are distinct molecular properties that determine the activity of prenylated (iso)flavonoids as antimicrobials and as potential EPIs, we compared the values of molecular descriptors of active EPIs with and without antimicrobial activity. For this comparison, we employed the molecular descriptors obtained from this study (prenylated (iso)flavonoids as potential NorA EPIs) and descriptors from the previous QSAR study for prenylated (iso)flavonoids as antimicrobials against MRSA, consisting of *vsurf_IW7* (hydrophilic integy moment), *h_pavgQ* (average total charge), *vsurf_CW3* (ratio between the hydrophilic surface and the total molecular surface at −1.0 kcal/mol), *PEOE_VSA_PPOS* (total positive polar van der Waals surface area), *E_vdw* (van der Waals surface energy), *vsurf_DD12* (contact distance of lowest and the second lowest hydrophobic energy points of a molecule), *PEOE_VSA+2* (sum of van der Waals surface area for each atom of which the partial charge is between +0.100e and +0.149e), and *vsurf_D4* (hydrophobic volume) (Kalli et al., 2021) (Supplementary Figure S6; Supplementary Table S6).

**FIGURE 5:**
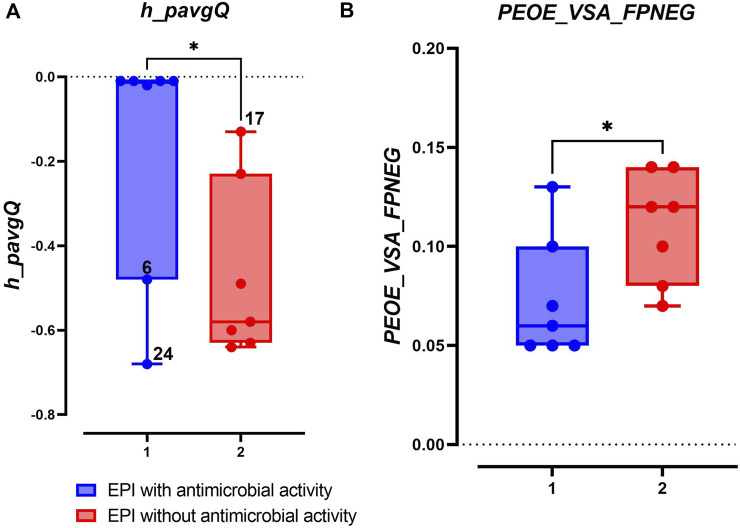
Two molecular descriptors, **(A)** *h_pavgQ* and **(B)** *PEOE_VSA_FPNEG*, that showed significant difference (*p* < 0.05) values between potential EPIs with and without antimicrobial activity. The descriptors’ values were obtained from the calculation of molecular descriptors (Supplementary Table S6). Compounds 6 and 24 that showed formal charges >0 are indicated in the figure.

Interestingly, there was a significant difference between potential EPIs with and without antimicrobial activity in terms of their formal charge at pH 7 (*h_pavgQ*) (Nguyen et al., 2021) and fractional negative polar van der Waals surface area (*PEOE_VSA_FPNEG*) (*p* < 0.05) (Figure 5). No significant differences were found with other studied descriptors (Supplementary Figure S6). Based on this comparison, potential EPIs with antimicrobial properties showed *h_pavgQ* values close to 0, except for compounds 7-*O*-prenylnaringenin (***6***) and licoisoflavone B (***24***), as shown in Figure 5A. Potential EPI compounds without antimicrobial properties demonstrated *h_pavgQ* values between −0.1 and −0.64 (Figure 5; Supplementary Table S6), which indicated that they have more negative formal charges than those with antimicrobial properties (*h* _*pavgQ* ∼ 0). In addition, potential EPIs with antimicrobial properties demonstrated a lower negative polar surface area fraction than those without antimicrobial properties (Figure 5B). Based on this study, we hypothesize the following: (i) formal charges and fractional negative polar surface area are key molecular properties that differentiate potential EPI and antimicrobial prenylated (iso)flavonoids; and (ii) a higher fractional negative polar surface area and more negative formal charges help to reduce the antimicrobial action of potential EPIs for this family of compounds. Overall, this comparison provides insight into the key molecular properties that can be useful for selecting prenylated (iso) flavonoids as potential NorA EPIs without having antimicrobial properties as an EPI is preferably a different chemical entity from an antimicrobial to prevent the risk of resistance (Sharma et al., 2019).

### 3.5 Prospects to improve the activity of prenylated (iso)flavonoids as NorA EPIs

To gain insights into possible structural modifications to improve the potency of prenylated (iso)flavonoids as potential NorA EPI, the seven best prenylated (iso)flavonoids were compared with a known NorA EPI that showed better activity than prenylated (iso)flavonoids. The synthetic NorA EPI PQQ16P was previously reported, and it exhibited 8-fold ciprofloxacin MIC reduction at 1.85 µM, i.e., five times lower concentration than the tested prenylated (iso)flavonoids and reserpine (Felicetti et al., 2018; Palazzotti et al., 2023). In addition, PQQ16P had low antimicrobial activity (MIC >200 µM) and low cytotoxicity (Palazzotti et al., 2023). Interestingly, PQQ16P has some similarities with prenylated (iso)flavonoids regarding the structural features, such as (i) the presence of aromatic rings, (ii) the presence of oxygen and nitrogen (as hydrogen bond acceptors) in the aromatic and heterocyclic ring, and (iii) the propoxy (O-alkyl) group in PQQ16P resembles the *O*-prenyl group present in some prenylated (iso)flavonoids used in this study (PQQ16P structure is shown in Supplementary Figure S1).

**FIGURE 6:**
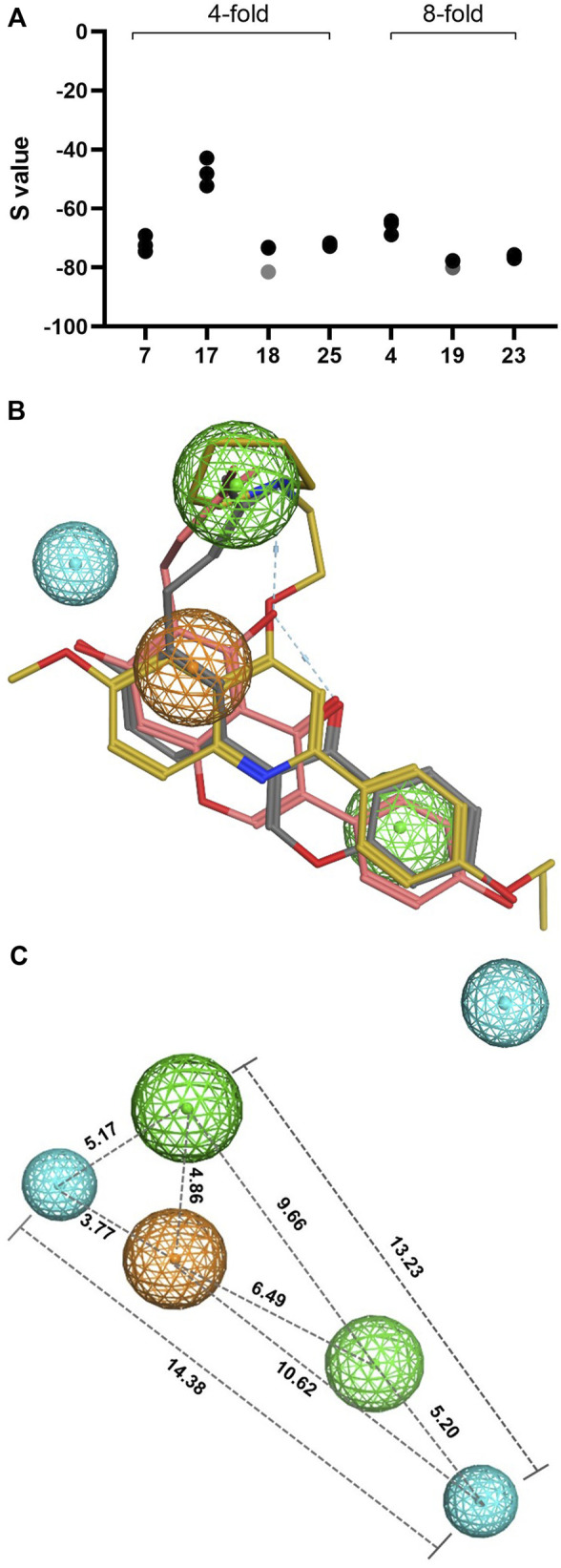
**(A)** Alignment scores (S value, y-axis) of prenylated (iso)flavonoids (indicated in numbers, x-axis) with PQQ16P from three repetitions. The 4-fold and 8-fold antibiotic potentiation (FR_CIP_) are indicated in the figure. The S values (calculated in MOE) lower than −80 and higher than −80 are represented by gray and black dots, respectively. **(B)** Pharmacophore elucidation of neobavaisoflavone (dark gray) and wighteone (pink) with the known EPI PQQ16P (yellow). The color of the spheres represents the following features: green spheres represent the hydrophobic features, and orange and blue spheres represent aromatic rings and hydrogen-bond acceptors, respectively. **(C)** Pharmacophore model showing the distances (in numbers) between the molecular features in Ångström.

To visualize and evaluate these structural similarities, the seven best prenylated (iso)flavonoids, namely, 8-prenylnaringenin (***4***), 6-*C*,7-*O*-diperenylnaringenin (***7***), glabrene (***17***), neobavaisoflavone (***18***), wighteone (***19***), licoisoflavone A (***23***), and glycyrrhisoflavone (***25***), were aligned or superposed with PQQ16P (Figure 6A). Based on the alignment scores (the lower the S value, the better the alignment), neobavaisoflavone (***18***) and wighteone (***23***) showed the best alignment (with S values of −81.50 and −80.06, respectively). The rest of the prenylated compounds demonstrated S scores between −60 and −80, except for glabrene (***17***), which showed S values between −40 and −60 (Figure 6A; Supplementary Table S7).

To further highlight the similarities in the structural features, pharmacophoric features were extracted from the best-aligned prenylated (iso)flavonoids (neobavaisoflavone and wighteone) and PQQ16P using pharmacophore query in MOE. Based on the pharmacophore analysis, neobavaisoflavone and wighteone shared similarities with PQQ16P, consisting of two hydrophobic features (green), one aromatic ring feature (orange), and two hydrogen bond acceptor features (blue) (Figures 6B,C), which were previously reported as essential chemical features for NorA recognition and binding (Palazzotti et al., 2023).

As observed in Figure 6B, the -OH groups in the A-ring of neobavaisoflavone and the B-ring of wighteone overlay with the propoxy (*O*-alkyl) group of PQQ16P, whereas the -OH groups in the B-ring of neobavaisoflavone and the A-ring of wighteone align with the *O*-methyl (-OCH_3_) group of PQQ16P. Moreover, the prenyl group in both prenylated isoflavonoids and the piperidine group of PQQ16P were pointed in a similar direction. It is important to consider that the positive ionizable piperidine group in PQQ16P is mainly responsible for electrostatic interaction with negatively charged residues in the NorA-binding pocket (e.g., Glu222 and Asp307) (Palazzotti et al., 2023). The lack of a positive ionizable group in prenylated (iso)flavonoids makes this type of interaction impossible.

Reflecting on these findings, prenylated (iso)flavonoids, mainly neobavaisoflavone and wighteone, are a good starting point for further structural optimization. Based on the structural alignment, possible modifications might be considered, such as *O*-prenylation, *O*-methylation, or the addition of a positive ionizable group, as observed in PQQ16P. Overall, our structural alignment and pharmacophore analysis with PQQ16P can further expand the possibility of improving the activity of prenylated (iso)flavonoids as potential NorA as EPIs.

## 4 Conclusion

In this study, the evaluation and QSAR analysis of prenylated (iso)flavonoids were conducted to understand their molecular features as potential NorA EPIs. Binary QSAR models were developed with a total prediction accuracy of up to 90% for active and 88% for inactive compounds. These models indicate that the fractional polar surface area, the balance of hydrophobicity–hydrophilicity, and the overall hydrophobicity were the main properties related to the potential NorA EPI activity. Furthermore, differences in negative formal charge and fractional negative polar surface area appeared to be important in distinguishing between the antimicrobial and potential NorA EPI activity of prenylated (iso)flavonoids. Compounds 8-prenylnaringenin (***4***), 6-*C*,7-*O*-diprenylnaringenin (***7***), wighteone (***19***), licoisoflavone A (***23***), and glycyrrhisoflavones (***25***) showed promising activity at 10 μM, in addition to the previously reported candidates neobavaisoflavone and glabrene. Among these compounds, 8-prenylnaringenin, wighteone, and licoisoflavone A at a concentration of 10 µM (≤¼ MIC) showed the best potentiation effect, causing up to 8-fold ciprofloxacin MIC reduction.

Based on our findings, there appears to be a window of opportunity for prenylated (iso)flavonoids as potential NorA EPIs without having antimicrobial activity. The structural alignment and the similar pharmacophoric features of prenylated (iso)flavonoids and the known NorA EPI PQQ16P further indicated that prenylated (iso)flavonoids, mainly neobavaisoflavone and wighteone, might be amenable for further enhancing their activity as potential NorA EPIs. In conclusion, this research provides new insight into NorA EPI discovery by proposing new potential EPIs and unraveling the key molecular features of prenylated (iso)flavonoids as potential NorA inhibitors.

## Data Availability

The original contributions presented in the study are included in the article/Supplementary Material, further inquiries can be directed to the corresponding author.
