# Supplementary material for: Molecular features of prenylated (iso)flavonoids from Fabaceae in relation to their potential NorA inhibition in Staphylococcus aureus
Source: Front Pharmacol. 2026 Jan 30;16:1715533. doi: 10.3389/fphar.2025.1715533 (PMC12903279; doi:10.3389/fphar.2025.1715533)
Supplement: Supplementary file 1 [file DataSheet1.pdf]

Molecular features of prenylated (iso)flavonoids from Fabaceae in relation to their potential NorA inhibition in *Staphylococcus aureus*

Marina Ika Irianti[1,2], Janniek Henrieke Ritsema[1], Jos Hageman[3], Jean-Paul Vincken[1], Carla Araya-Cloutier[1]*

[1]Laboratory of Food Chemistry, Wageningen University and Research, Bornse Weilanden, 6708 PD, Wageningen, The Netherlands

[2]Laboratory of Microbiology and Biotechnology, Faculty of Pharmacy, Universitas Indonesia, 16424, Depok, Indonesia

[3]Biometris, Applied Statistics, Wageningen University and Research, Droevendaalsesteeg 1, 6708 PB, Wageningen, The Netherlands

*** Correspondence:**
Carla Araya-Cloutier,
carla.arayacloutier@wur.nl

SUPPLEMENTARY INFORMATION

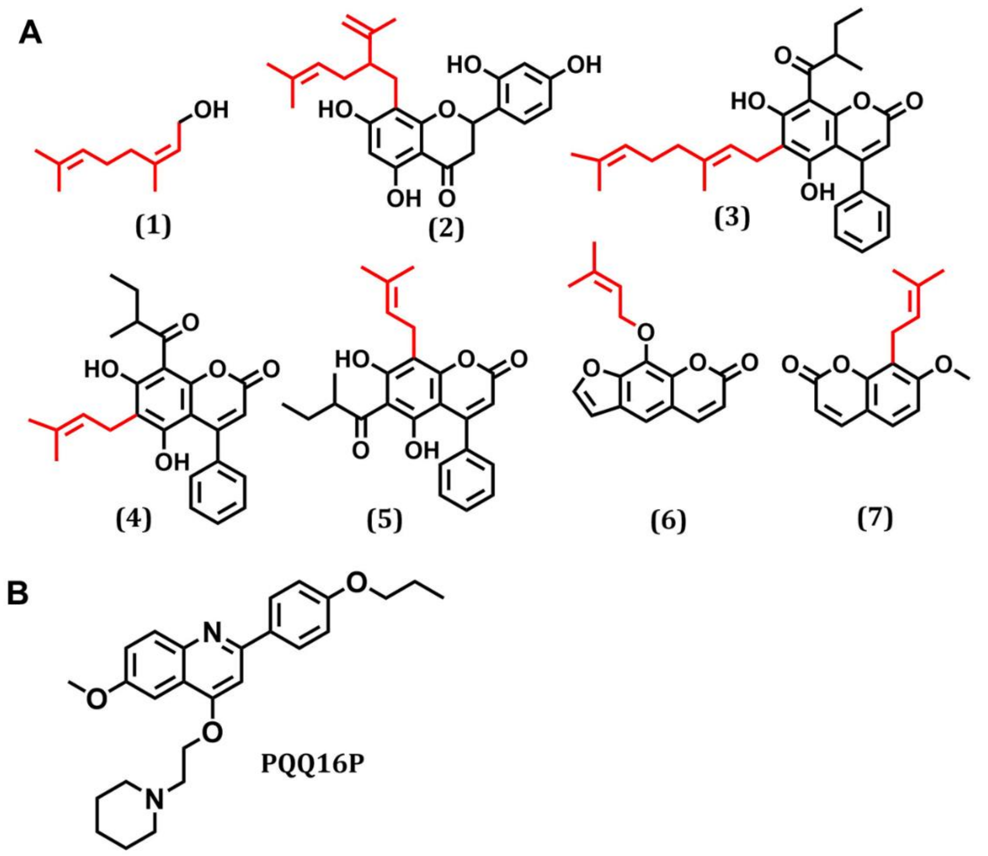

Figure S 1. (**A**) Structures of reported NorA EPIs with prenyl substitutions (1) nerol, (2) sophoraflavanone G, (3) 5,7-dihydroxy-8-(2-methylbutanoyl)-6-[3,7-dimethylocta-2,6-dienyl]-4-phenyl-2H-chromen-2-one, (4) 5,7-dihydroxy-6-(2-methyl-butanoyl)-8-(3-methylbut-2-enyl)-4-phenyl-2H-chromen-2-one, (5) 5,7-dihydroxy-8-(2-methylbutanoyl)-6-(3-methylbut-2-enyl)-4-phenyl-2H-chromen-2-one, (6) imperatorin, and (7) osthol. (**B**) the structure of PQQ16P.

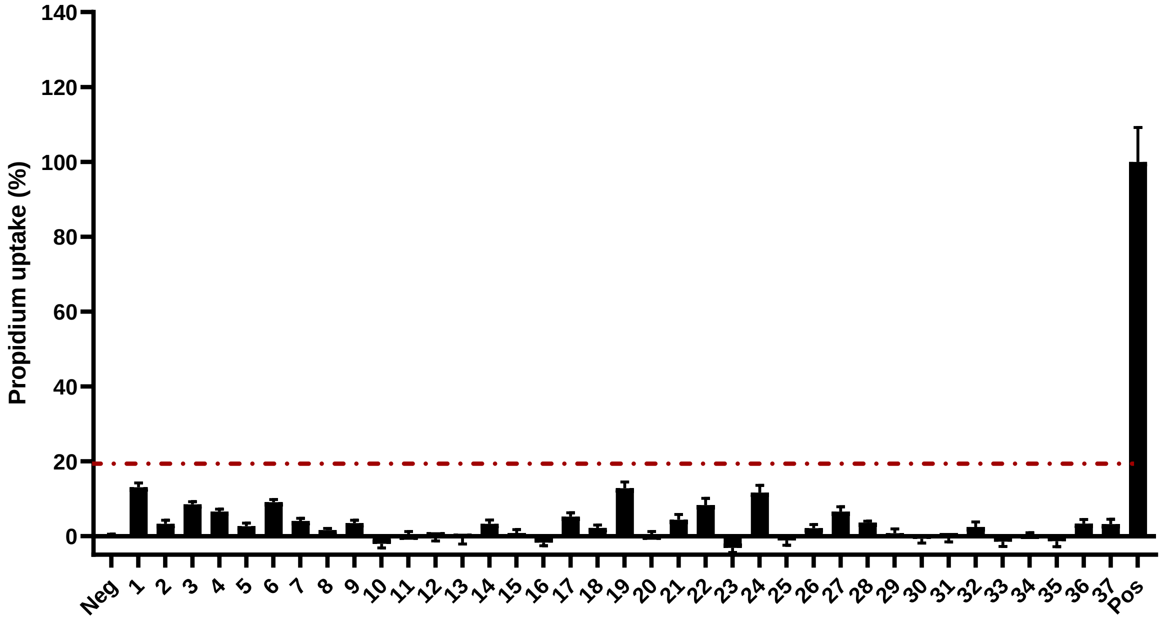

Figure S2. Propidium uptake of 37 prenylated (iso)flavonoids at concentration of 10 µM. The red dashed line indicates the threshold for the PI uptake, where the PI uptake below 20% is considered as no remarkable permeabilization effect. Neg and Pos refer to negative control and positive control, respectively. The propidium fluorescence emitting from untreated cells was set 0% in the negative control (Neg) and the maximum propidium uptake into heat-treated cells was set 100% in the positive control (Pos). The data shown are the percentage of propidium uptake from three biological replicates with standard error of the mean (SEM).

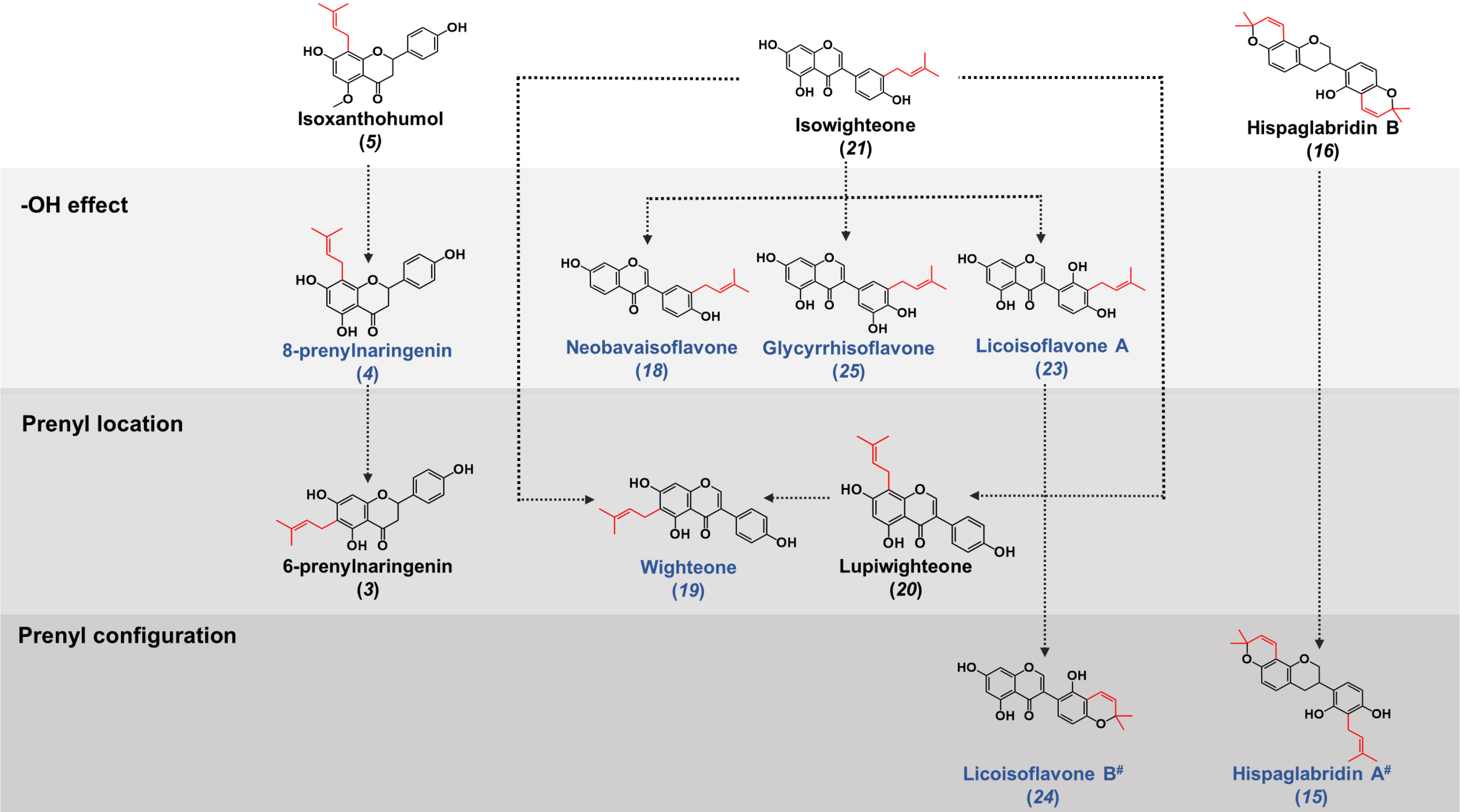

Figure S3. The structure-activity relationship (SAR) of prenylated (iso)flavonoids tested in this study. Non-EPI and EPI at a concentration of 10 μM are indicated in black and blue text, respectively.
# indicates EPI with antimicrobial activity.

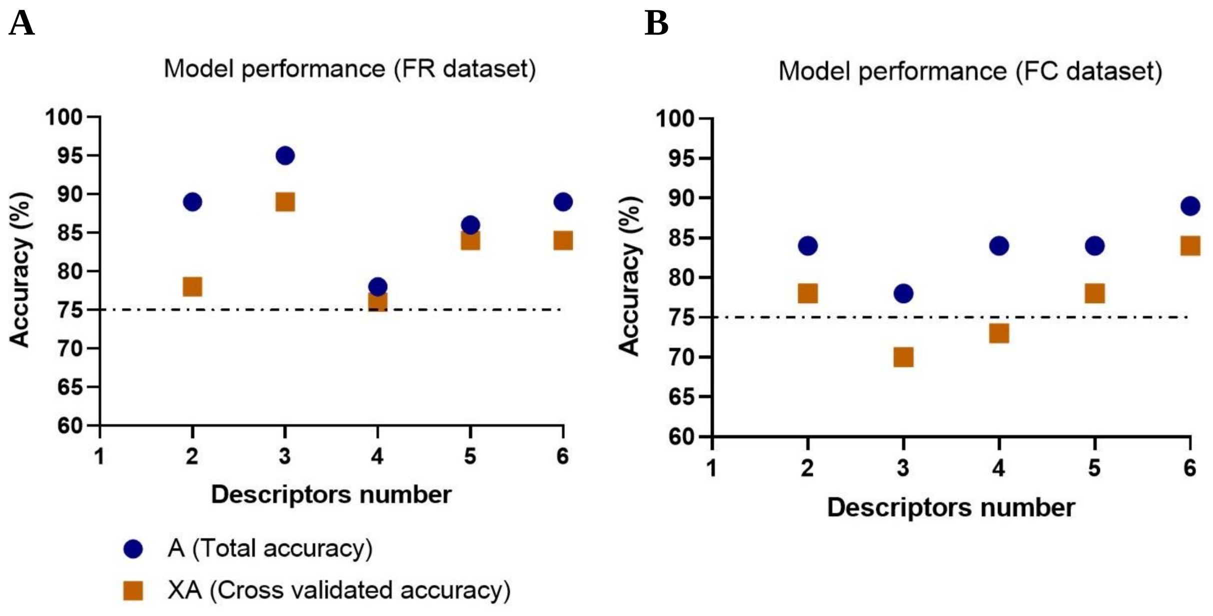

Figure S4. Model performances of binary QSAR models with 2-6 descriptors number using (**A**) FR_{CIP} dataset and (**B**) FC dataset.

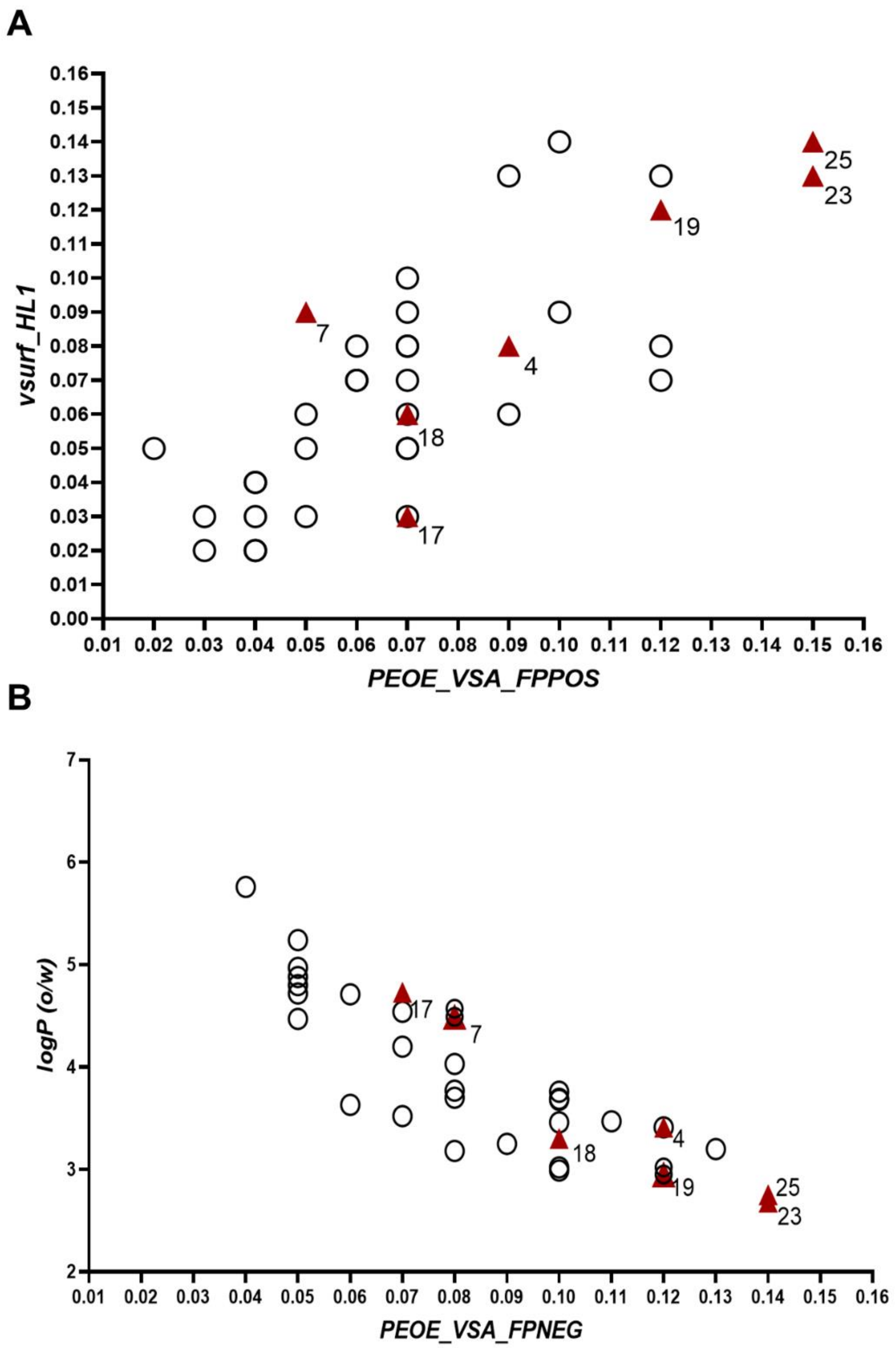

Figure S5. The range of descriptors' values from the QSAR models obtained from (**A**) FR_{CIP} dataset and (**B**) FC dataset. Red triangles showed active EPIs (without antimicrobial activity).

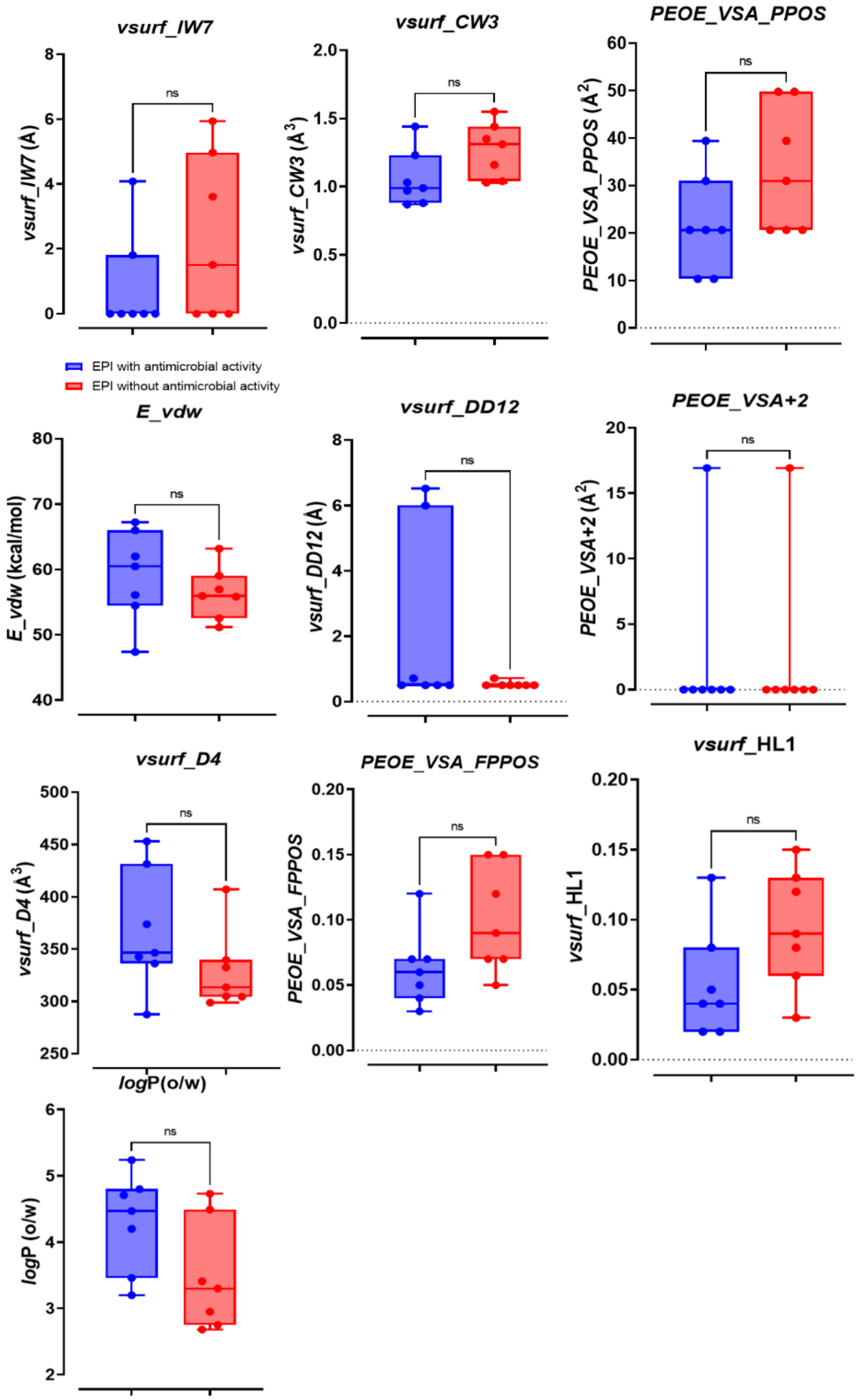

Figure S6. Comparison of molecular descriptors' values between EPIs with and without antimicrobial activity obtained in this study. Ns indicates no significant different between two groups.

Table S1. Purity of prenylated (iso)flavonoids used in this study. [a] ^{1}H NMR purity without considering residual solvents or additives from the purification process (i.e. tert-butanol and formic acid). [b] If the estimated ^{1}H NMR purity including tert-butanol and formic acid as impurity was ≤75 %, the content was corrected (compounds **12**, **22**, and **24**). [c] Reported by Ritsema *et al*. (2025), [d] van Dinteren *et al*. (2025), [e] van de Schans *et al*. (2015), [f] van de Schans *et al.* (2016). [g] Checked using the method reported by van Dinteren *et al*. (2021) to confirm their identities, as sometimes dehydroglyceollin III is also referred to as glyceollin VI in the literature. [h] Checked using the method reported by van de Schans *et al*. (2016) (unpublished data). N.a. means not available.

Subclass		Name	UV_{280} purity (%)	^{1}H NMR purity (%)	Supplier or Source
**Flavanones**					
	(***1***)	Bavachin	95	n.a.	Chemfaces (China)
	(***2***)	Isobavachin	94	n.a.	Chemfaces (China)
	(***3***)	6-prenylnaringenin	94	n.a.	Chemfaces (China)
	(***4***)	8-prenylnaringenin	92	95	Synthesized [c]
	(***5***)	Isoxanthohumol	93	n.a.	Chemfaces (China)
	(***6***)	7-*O*-prenylnaringenin	>95	>95	Synthesized [c]
	(***7***)	6-*C*,7-*O*-diprenylnaringenin	>95	>95	Synthesized [c]
	(***8***)	8-*C*,7-*O*-diprenylnaringenin	>95	>95	Synthesized [c]
	(***9***)	3'-*C*,7-*O*-diprenylnaringenin	>95	84	Synthesized [c]
	(***10***)	7,4'-*O*-diprenylnaringenin	>95	>95	Synthesized [c]
**Isoflavans**					
	(***11***)	4'-*O*-methylglabridin	>95	81	*Glycyrrhiza* spp. roots [e]
	(***12***)	Licorisoflavan A	82[a]	86 [a,b]	*Glycyrrhiza* spp. roots [d]
	(***13***)	Glabridin	>95	n.a.	Wako (Japan)
	(***14***)	Licoricidin	73[a]	94[a]	*Glycyrrhiza* spp. roots [d]
	(***15***)	Hispaglabridin A	86[a]	85[a]	*Glycyrrhiza* spp. roots [d]
	(***16***)	Hispaglabridin B	90	≥95	*Glycyrrhiza* spp. roots [e]
**Isoflavene**					
	(***17***)	Glabrene	85	n.a.	Arctom (USA)
**Isoflavone**					
	(***18***)	Neobavaisoflavone	≥95%	n.a.	PhytoLab (Germany)
	(***19***)	Wighteone	80	n.a.	Chemfaces (China)
	(***20***)	Lupiwighteone	84	n.a.	Chemfaces (China)
	(***21***)	Isowighteone	92	n.a.	Chemfaces (China)
	(***22***)	Glabrone	79[a]	66[a,b]	*Glycyrrhiza* spp. roots [d]
	(***23***)	Licoisoflavone A	86	n.a.	Chemfaces (China)
	(***24***)	Licoisoflavone B	85[a]	83[a,b]	*Glycyrrhiza* spp. Roots [d]
	(***25***)	Glycyrrhisoflavone	79	n.a.	Chemfaces (China)
	(***26***)	7-*O*-prenylgenistein	>95	>95	Synthesized [c]
	(***27***)	8-*C*,7-*O*-diprenylgenistein	>95	86	Synthesized [c]
**Pterocarpan**					
	(***28***)	Glyceollidin II	99[h]	>95	*Glycine max* (L.) Merrill [f]
	(***29***)	Glyceollin I	93[h]	90	*Glycine max* (L.) Merrill [f]
	(***30***)	Glyceollin II	90[h]	92	*Glycine max* (L.) Merrill [f]
	(***31***)	Glyceollin III	99[h]	>95	*Glycine max* (L.) Merrill [f]
	(***32***)	Glyceollin IV	99[h]	>95	*Glycine max* (L.) Merrill [f]
	(***33***)	Glyceollin VI	>95[g]	>95	*Glycine max* (L.) Merrill [f]
	(***34***)	Glyceofuran	95[h]	95	*Glycine max* (L.) Merrill [f]
**Pterocarpene**					
	(***35***)	Dehydroglyceollin I	85[h]	93	*Glycine max* (L.) Merrill [f]
	(***36***)	Dehydroglyceollin II	99[h]	>95	*Glycine max* (L.) Merrill [f]
	(***37***)	Dehydroglyceollin III	77[g]	>95	*Glycine max* (L.) Merrill [f]

Table S2. List of molecular descriptors used in the QSAR-binary study.

No.	Descriptors	No.	Descriptors	No.	Descriptors	No.	Descriptors	No.	Descriptors
1	AM1_dipole	41	FASA-	81	RPC-	121	vsurf_EWmin1	161	weinerPath
2	AM1_E	42	FASA_P	82	SlogP	122	vsurf_EWmin2	162	weinerPol
3	AM1_Eele	43	FCASA+	83	SlogP_VSA0	123	vsurf_EWmin3	163	zagreb
4	AM1_HF	44	FCASA-	84	SlogP_VSA4	124	vsurf_G		
5	AM1_IP	45	Kier1	85	SlogP_VSA5	125	vsurf_HB1		
6	AM1_LUMO	46	Kier3	86	SlogP_VSA7	126	vsurf_HB2		
7	ASA	47	KierFlex	87	SlogP_VSA9	127	vsurf_HB3		
8	ASA+	48	logP(o/w)	88	SMR_VSA3	128	vsurf_HB4		
9	ASA-	49	logS	89	SMR_VSA5	129	vsurf_HB5		
10	ASA_H	50	MNDO_dipole	90	SMR_VSA6	130	vsurf_HB6		
11	ASA_P	51	MNDO_HF	91	SMR_VSA7	131	vsurf_HB7		
12	a_donacc	52	MNDO_IP	92	std_dim1	132	vsurf_HL1		
13	a_hyd	53	MNDO_LUMO	93	std_dim2	133	vsurf_HL2		
14	a_IC	54	npr1	94	std_dim3	134	vsurf_ID1		
15	a_ICM	55	npr2	95	TPSA	135	vsurf_ID2		
16	a_nH	56	opr_brigid	96	VDistEq	136	vsurf_ID3		
17	balabanJ	57	opr_nrot	97	vdw_area	137	vsurf_ID4		
18	bpol	58	PC-	98	vdw_vol	138	vsurf_ID6		
19	b_count	59	PEOE_VSA+0	99	vsa_acc	139	vsurf_ID7		
20	b_heavy	60	PEOE_VSA+1	100	vsa_hyd	140	vsurf_ID8		
21	b_rotN	61	PEOE_VSA-0	101	vsa_pol	141	vsurf_IW1		
22	b_single	62	PEOE_VSA-1	102	vsurf_A	142	vsurf_IW2		
23	CASA+	63	PEOE_VSA-6	103	vsurf_CP	143	vsurf_IW3		
24	CASA-	64	PEOE_VSA_FPNEG	104	vsurf_CW1	144	vsurf_IW4		
25	DASA	65	PEOE_VSA_FPOL	105	vsurf_CW2	145	vsurf_IW5		
26	DCASA	66	PEOE_VSA_FPOS	106	vsurf_CW3	146	vsurf_IW6		
27	density	67	PEOE_VSA_FPPOS	107	vsurf_CW4	147	vsurf_IW7		
28	diameter	68	PEOE_VSA_NEG	108	vsurf_CW7	148	vsurf_R		
29	dipole	69	PEOE_VSA_PNEG	109	vsurf_D1	149	vsurf_W1		
30	E_ang	70	PEOE_VSA_POL	110	vsurf_D6	150	vsurf_W2		
31	E_ele	71	PEOE_VSA_POS	111	vsurf_D7	151	vsurf_W3		
32	E_nb	72	PEOE_VSA_PPOS	112	vsurf_D8	152	vsurf_W4		
33	E_oop	73	PM3_dipole	113	vsurf_DD12	153	vsurf_W5		
34	E_sol	74	PM3_HF	114	vsurf_DD13	154	vsurf_W6		
35	E_stb	75	PM3_IP	115	vsurf_DD23	155	vsurf_Wp1		
36	E_str	76	PM3_LUMO	116	vsurf_DW12	156	vsurf_Wp2		
37	E_strain	77	pmi	117	vsurf_DW13	157	vsurf_Wp3		
38	E_tor	78	pmi1	118	vsurf_DW23	158	vsurf_Wp4		
39	E_vdw	79	pmi2	119	vsurf_EDmin1	159	vsurf_Wp5		
40	FASA+	80	RPC+	120	vsurf_EDmin3	160	vsurf_Wp6		

Table S3. Performance of binary QSAR models with two descriptors by using (**A**) FR_{CIP} dataset and (**B**) FC dataset with a component limit of 2-5 and smooth 0.08-0.25. The best models (highlighted in grey) were selected based on the overall performance (accuracies) and the most interpretable descriptors.

A. FR_{CIP} dataset

Component limit		Smooth																	
		**0.08**	**0.09**	**0.1**	**0.11**	**0.12**	**0.13**	**0.14**	**0.15**	**0.16**	**0.17**	**0.18**	**0.19**	**0.2**	**0.21**	**0.22**	**0.23**	**0.24**	**0.25**
**2**	**A**	0.81	0.81	0.81	0.78	0.78	0.81	0.78	0.76	0.76	0.76	0.76	0.76	0.76	0.76	0.73	0.76	0.7	0.7
	**A1**	0.76	0.76	0.9	0.9	0.9	0.81	0.76	0.86	0.86	0.86	0.67	0.67	0.67	0.81	0.81	0.86	0.86	0.86
	**A0**	0.88	0.88	0.69	0.63	0.63	0.81	0.81	0.63	0.63	0.63	0.88	0.88	0.88	0.69	0.63	0.63	0.5	0.5
	**XA**	0.68	0.68	0.68	0.68	0.68	0.68	0.68	0.73	0.73	0.73	0.7	0.7	0.7	0.62	0.57	0.65	0.62	0.65
	**XA1**	0.62	0.62	0.81	0.81	0.81	0.57	0.57	0.81	0.81	0.81	0.57	0.57	0.57	0.67	0.57	0.71	0.76	0.81
	**XA0**	0.75	0.75	0.5	0.5	0.5	0.81	0.81	0.63	0.63	0.63	0.88	0.88	0.88	0.56	0.56	0.56	0.44	0.44
**3**	**A**	0.86	0.84	0.84	0.84	0.84	0.84	0.86	**0.89**	**0.89**	0.89	0.89	0.89	0.76	0.73	0.73	0.73	0.78	0.78
	**A1**	0.81	0.76	0.76	0.76	0.76	0.76	0.81	**0.9**	**0.9**	0.9	0.9	0.9	0.76	0.71	0.71	0.71	0.76	0.76
	**A0**	0.94	0.94	0.94	0.94	0.94	0.94	0.94	**0.88**	**0.88**	0.88	0.88	0.88	0.75	0.75	0.75	0.75	0.81	0.81
	**XA**	0.84	0.84	0.84	0.84	0.84	0.84	0.78	**0.84**	**0.84**	0.81	0.78	0.76	0.68	0.68	0.68	0.65	0.70	0.7
	**XA1**	0.76	0.76	0.76	0.76	0.76	0.76	0.67	**0.81**	**0.81**	0.81	0.81	0.76	0.67	0.67	0.67	0.62	0.62	0.62
	**XA0**	0.94	0.94	0.94	0.94	0.94	0.94	0.94	**0.88**	**0.88**	0.81	0.75	0.75	0.69	0.69	0.69	0.69	0.81	0.81
**4**	**A**	0.86	0.86	0.84	0.81	0.81	0.81	0.81	0.81	0.81	0.81	0.81	0.78	0.78	0.78	0.78	0.78	0.78	0.76
	**A1**	0.86	0.81	0.76	0.71	0.71	0.71	0.71	0.71	0.71	0.71	0.71	0.71	0.71	0.71	0.71	0.71	0.71	0.67
	**A0**	0.88	0.94	0.94	0.94	0.94	0.94	0.94	0.94	0.94	0.94	0.94	0.88	0.88	0.88	0.88	0.88	0.88	0.88
	**XA**	0.78	0.81	0.81	0.78	0.78	0.78	0.76	0.76	0.76	0.76	0.73	0.73	0.73	0.73	0.73	0.73	0.73	0.73
	**XA1**	0.76	0.76	0.76	0.71	0.71	0.71	0.71	0.71	0.71	0.71	0.67	0.67	0.67	0.67	0.67	0.67	0.62	0.62
	**XA0**	0.81	0.88	0.88	0.88	0.88	0.88	0.81	0.81	0.81	0.81	0.81	0.81	0.81	0.81	0.81	0.81	0.88	0.88
**5**	**A**	0.86	0.86	0.86	0.86	0.89	0.81	0.81	0.81	0.76	0.81	0.81	0.81	0.81	0.81	0.81	0.78	0.78	0.76
	**A1**	0.81	0.81	0.81	0.81	0.9	0.71	0.71	0.76	0.81	0.76	0.76	0.76	0.76	0.76	0.76	0.71	0.71	0.67
	**A0**	0.94	0.94	0.94	0.94	0.88	0.94	0.94	0.88	0.69	0.88	0.88	0.88	0.88	0.88	0.88	0.88	0.88	0.88
	**XA**	0.76	0.76	0.76	0.76	0.89	0.78	0.76	0.76	0.73	0.73	0.7	0.73	0.7	0.7	0.7	0.73	0.73	0.73
	**XA1**	0.67	0.67	0.67	0.71	0.9	0.71	0.71	0.76	0.81	0.76	0.71	0.71	0.67	0.67	0.67	0.67	0.62	0.62
	**XA0**	0.88	0.88	0.88	0.81	0.88	0.88	0.81	0.75	0.63	0.69	0.69	0.75	0.75	0.75	0.75	0.81	0.88	0.88

B. FC dataset

Component limit		Smooth																	
		**0.08**	**0.09**	**0.1**	**0.11**	**0.12**	**0.13**	**0.14**	**0.15**	**0.16**	**0.17**	**0.18**	**0.19**	**0.2**	**0.21**	**0.22**	**0.23**	**0.24**	**0.25**
**2**	**A**	0.81	0.81	0.81	0.81	0.81	0.81	0.78	0.78	0.78	0.76	0.73	0.73	0.81	0.81	0.81	0.76	0.76	0.76
	**A1**	0.78	0.78	0.61	0.61	0.61	0.61	0.61	0.61	0.61	0.56	0.5	0.5	0.72	0.72	0.72	0.56	0.56	0.56
	**A0**	0.84	0.84	1	1	1	1	0.95	0.95	0.95	0.95	0.95	0.95	0.89	0.89	0.89	0.95	0.95	0.95
	**XA**	0.76	0.76	0.68	0.68	0.68	0.68	0.65	0.65	0.65	0.65	0.62	0.62	0.78	0.78	0.78	0.57	0.54	0.54
	**XA1**	0.72	0.72	0.56	0.56	0.56	0.56	0.5	0.5	0.5	0.5	0.5	0.5	0.67	0.67	0.67	0.5	0.5	0.5
	**XA0**	0.79	0.79	0.79	0.79	0.79	0.79	0.79	0.79	0.79	0.79	0.74	0.74	0.89	0.89	0.89	0.63	0.58	0.58
**3**	**A**	0.81	0.81	0.76	0.81	0.81	0.81	0.81	0.81	0.84	0.76	0.78	0.84	0.81	0.78	0.78	0.78	0.78	0.65
	**A1**	0.72	0.72	0.67	0.72	0.72	0.72	0.72	0.72	0.78	0.67	0.67	0.83	0.67	0.67	0.67	0.67	0.67	0.39
	**A0**	0.89	0.89	0.84	0.89	0.89	0.89	0.89	0.89	0.89	0.84	0.89	0.84	0.95	0.89	0.89	0.89	0.89	0.89
	**XA**	0.76	0.76	0.73	0.76	0.78	0.78	0.78	0.76	0.73	0.68	0.7	0.76	0.7	0.68	0.65	0.68	0.7	0.65
	**XA1**	0.67	0.61	0.61	0.72	0.67	0.67	0.67	0.67	0.61	0.56	0.56	0.67	0.61	0.61	0.56	0.5	0.67	0.39
	**XA0**	0.84	0.89	0.84	0.79	0.89	0.89	0.89	0.84	0.84	0.79	0.84	0.84	0.79	0.74	0.74	0.84	0.74	0.89
**4**	**A**	0.76	0.7	0.86	0.84	0.81	**0.84**	0.7	0.76	0.76	0.73	0.81	0.78	0.78	0.76	0.76	0.76	0.76	0.76
	**A1**	0.67	0.5	0.83	0.67	0.67	**0.83**	0.61	0.67	0.78	0.72	0.72	0.67	0.67	0.67	0.67	0.67	0.61	0.61
	**A0**	0.84	0.89	0.89	1	0.95	**0.84**	0.79	0.84	0.74	0.74	0.89	0.89	0.89	0.84	0.84	0.84	0.89	0.89
	**XA**	0.65	0.62	0.73	0.62	0.54	**0.78**	0.62	0.68	0.68	0.68	0.7	0.73	0.73	0.68	0.68	0.65	0.7	0.68
	**XA1**	0.44	0.44	0.72	0.44	0.5	**0.78**	0.5	0.5	0.72	0.61	0.61	0.61	0.61	0.56	0.56	0.5	0.56	0.56
	**XA0**	0.84	0.79	0.74	0.79	0.58	**0.79**	0.74	0.84	0.63	0.74	0.79	0.84	0.84	0.79	0.79	0.79	0.84	0.79
**5**	**A**	0.7	0.76	0.78	0.81	0.76	0.78	0.81	0.86	0.76	0.7	0.68	0.78	0.78	0.78	0.73	0.68	0.68	0.7
	**A1**	0.61	0.67	0.72	0.72	0.67	0.67	0.72	0.78	0.67	0.61	0.56	0.72	0.67	0.67	0.56	0.56	0.5	0.72
	**A0**	0.79	0.84	0.84	0.89	0.84	0.89	0.89	0.95	0.84	0.79	0.79	0.84	0.89	0.89	0.89	0.79	0.84	0.68
	**XA**	0.59	0.59	0.78	0.76	0.65	0.68	0.73	0.78	0.68	0.68	0.68	0.73	0.73	0.73	0.59	0.59	0.62	0.54
	**XA1**	0.44	0.5	0.72	0.67	0.56	0.44	0.61	0.72	0.56	0.56	0.56	0.61	0.61	0.61	0.44	0.44	0.44	0.55
	**XA0**	0.74	0.68	0.84	0.84	0.74	0.89	0.84	0.84	0.79	0.79	0.79	0.84	0.84	0.84	0.74	0.74	0.79	0.53

Table S4. Checkerboard results of 37 prenylated (iso)flavonoids in the presence of ciprofloxacin in *norA* overexpressing *S. aureus*, performed in three biological repetitions.

Subclass	Name	1st Bio								2nd Bio								3rd Bio							
		MIC of ciprofloxacin in combination with prenylated isoflavonoids (μM)						Reduction (n-fold) Cip MIC at 10 μM (FR)	MIC compounds alone (μM)	MIC of ciprofloxacin in combination with prenylated isoflavonoids (μM)						Reduction (n-fold) Cip MIC at 10 μM (FR)	MIC compounds alone (μM)	MIC of ciprofloxacin in combination with prenylated isoflavonoids (μM)						Reduction (n-fold) Cip MIC at 10 μM (FR)	MIC compounds alone (μM)
		40	20	10	5	2,5	0			40	20	10	5	2,5	0			40	20	10	5	2,5	0		
**Flavanones**																									
(**1**)	Bavachin	3	3	6	12	24	24	4,00	>40	3	3	6	12	12	24	4,00	>40	3	3	6	12	24	24	4,00	>40
(**2**)	Isobavachin	3	6	12	24	24	48	4,00	>40	1,5	3	12	12	24	24	2,00	>40	3	6	12	24	24	48	4,00	>40
(**3**)	6-prenylnaringenin	0	3	6	12	12	48	8,00	40	0	3	6	12	12	24	4,00	40	0	3	6	12	12	48	8,00	40
(**4**)	8-prenylnaringenin	0	3	6	12	24	48	8,00	40	0	3	6	12	24	24	4,00	40	0	3	6	12	24	48	8,00	40
(**5**)	Isoxanthohumol	6	12	24	24	24	48	2,00	>40	3	12	12	24	24	24	2,00	>40	6	12	24	24	24	48	2,00	>40
(**6**)	7-*O*-prenylnaringenin	0	0	12	24	24	48	4,00	20	0	0	12	24	24	24	2,00	20	0	0	12	24	24	48	4,00	20
(**7**)	6-*C*, 7-*O*-diprenylnaringenin	12	12	12	24	24	48	4,00	>40	12	12	12	24	24	24	2,00	>40	12	12	24	24	24	48	2,00	>40
(**8**)	8-*C*, 7-*O*-diprenylnaringenin	24	24	24	24	24	48	2,00	>40	12	24	24	24	24	24	1,00	>40	24	24	24	24	24	48	2,00	>40
(**9**)	3'-*C*, 7-*O*-diprenylnaringenin	24	24	24	24	24	48	2,00	>40	24	24	24	24	24	24	1,00	>40	24	24	24	24	24	48	2,00	>40
(**10**)	7,4'-*O*-diprenylnaringenin	24	24	24	24	24	48	2,00	>40	24	24	24	24	24	24	1,00	>40	24	24	24	24	24	48	2,00	>40
**Isoflavans**																									
(**11**)	4'-*O*-methylglabridin	0	0	6	12	24	48	8,00	20	0	0	6	12	24	24	4,00	20	0	0	6	12	24	48	8,00	20
(**12**)	Licorisoflavan A	12	12	24	24	24	48	2,00	>40	12	12	24	24	24	24	1,00	>40	12	12	24	24	24	48	2,00	>40
(**13**)	Glabridin	0	0	12	24	24	48	4,00	20	0	0	12	24	24	24	2,00	20	0	3	12	24	24	48	4,00	40
(**14**)	Licoricidin	0	0	3	12	24	48	16,00	20	0	0	3	12	24	24	8,00	20	0	0	6	12	24	48	8,00	20
(**15**)	Hispaglabridin A	0	0	12	12	24	48	4,00	20	0	0	6	12	24	24	4,00	20	0	1,5	12	12	24	48	4,00	40
(**16**)	Hispaglabridin B	6	6	12	24	24	24	2,00	40	0	6	12	24	24	24	2,00	40	0	6	12	24	24	24	2,00	40
**Isoflavene**																									
(**17**)	Glabrene	1,5	3	6	12	24	24	4,00	>40	0	3	6	12	24	24	4,00	>40	1,5	3	6	12	24	24	4,00	>40
**Isoflavone**																									
(**18**)	Neobavaisoflavone	0	3	6	12	24	24	4,00	40	0	3	6	12	24	24	4,00	40	0	3	6	12	24	24	4,00	40
(**19**)	Wighteone	0	3	6	12	12	24	4,00	40	0	3	6	12	12	24	4,00	40	0	3	6	12	24	48	8,00	40
(**20**)	Lupiwighteone	6	12	12	12	24	24	2,00	>40	6	12	12	24	24	24	2,00	>40	6	12	12	24	24	24	2,00	>40
(**21**)	Isowighteone	6	6	12	12	12	24	2,00	>40	6	12	12	12	12	24	2,00	>40	12	12	12	12	12	24	2,00	>40
(**22**)	Glabrone	0	3	6	6	12	48	8,00	40	0	3	6	12	12	24	4,00	40	0	3	6	12	12	24	4,00	40
(**23**)	Licoisoflavone A	0	3	6	12	24	48	8,00	40	0	3	6	12	24	48	8,00	40	0	3	6	12	24	48	8,00	40
(**24**)	Licoisoflavone B	0	0	3	6	12	48	16,00	20	0	0	3	6	12	24	8,00	20	0	0	3	6	12	24	8,00	20
(**25**)	Glycyrrhisoflavone	3	6	12	24	24	48	4,00	>40	3	6	24	24	48	48	2,00	>40	1,5	3	12	12	24	24	2,00	>40
(**26**)	7-*O*-prenylgenistein	24	24	24	24	24	48	2,00	>40	24	24	24	24	24	24	1,00	>40	24	24	24	24	24	24	1,00	>40
(**27**)	8-*C*, 7-*O*-diprenylgenistein	24	24	24	24	24	24	1,00	>40	24	24	24	24	24	48	2,00	>40	24	24	24	24	24	24	1,00	>40
**6a-OH-Pterocarpan**																									
(**28**)	Glyceollidin II	6	12	24	24	24	48	2,00	>40	12	24	24	24	24	24	1,00	>40	6	12	24	24	24	24	1,00	>40
(**29**)	Glyceollin I	24	24	24	24	24	48	2,00	>40	24	24	24	24	24	48	2,00	>40	24	24	24	24	24	24	1,00	>40
(**30**)	Glyceollin II	12	24	24	48	48	48	2,00	>40	12	24	24	24	24	48	2,00	>40	12	24	24	24	24	24	1,00	>40
(**31**)	Glyceollin III	12	24	24	48	48	48	2,00	>40	12	24	24	24	24	48	2,00	>40	12	24	24	24	24	48	2,00	>40
(**32**)	Glyceollin IV	6	12	24	24	24	48	2,00	>40	6	12	24	24	24	48	2,00	>40	6	12	24	24	24	24	1,00	>40
(**33**)	Glyceollin VI	6	12	24	24	24	48	2,00	>40	6	12	12	24	24	48	4,00	>40	6	12	24	24	24	48	2,00	>40
(**34**)	Glyceofuran	24	24	48	48	48	48	1,00	>40	24	24	24	24	24	24	1,00	>40	24	24	24	24	24	24	1,00	>40
**6a,11a-Pterocarpene**																									
(**35**)	Dehydroglyceollin I	0	0	12	24	24	48	4,00	20	0	0	12	24	24	24	2,00	20	0	0	12	24	24	48	4,00	20
(**36**)	Dehydroglyceollin II	0	0	12	24	24	48	4,00	20	0	0	12	24	24	24	2,00	20	0	0	12	24	24	24	2,00	20
(**37**)	Dehydroglyceollin III	3	12	12	24	24	48	4,00	>40	3	6	12	12	24	24	2,00	>40	6	12	12	24	24	24	2,00	>40
	Reserpine	12	6	12	24	24	24	2,00	>40	12	6	12	24	24	24	2,00	>40	12	12	12	24	24	48	4,00	>40

Table S5. Molecular properties of 37 prenylated (iso)flavonoids from QSAR-binary models with FR_{CIP} and FC dataset. All the values for each descriptor were generated by MOE.

**No.**	**Subclass**	**Compound**	**EPI/ non-EPI at 10 µM**	**Single/ double prenylation**	**Prenyl configuration**	***PEOE_VSA _FPPOS***	***vsurf_HL1***	***PEOE_VSA_ FPNEG***	***log*P(o/w)**
**Flavanones**									
(*1*)		Bavachin	Non-EPI	Single	Chain	0.07	0.07	0.10	3.76
(*2*)		Isobavachin	Non-EPI	Single	Chain	0.07	0.06	0.10	3.69
(*3*)		6-prenylnaringenin	Non-EPI	Single	Chain	0.09	0.13	0.12	3.41
(*4*)		8-prenylnaringenin	**EPI**	Single	Chain	0.09	0.08	0.12	3.41
(*5*)		Isoxanthohumol	Non-EPI	Single	Chain	0.06	0.07	0.10	3.68
(*6*)		7-*O*-prenylnaringenin	EPI-Ant	Single	Chain	0.06	0.07	0.10	3.46
(*7*)		6-*C*,7-*O*-diprenylnaringenin	**EPI**	Double	Chain	0.05	0.09	0.08	4.49
(*8*)		8-*C*,7-*O*-diprenylnaringenin	Non-EPI	Double	Chain	0.05	0.06	0.08	4.49
(*9*)		3'-*C*,7-*O*-diprenylnaringenin	Non-EPI	Double	Chain	0.05	0.05	0.08	4.57
(*10*)		7,4'-*O*-diprenylnaringenin	Non-EPI	Double	Chain	0.02	0.05	0.07	4.54
**Isoflavans**									
(*11*)		4'-*O*-methylglabridin	EPI-Ant	Single	Ring	0.03	0.02	0.05	4.47
(*12*)		Licorisoflavan A	Non-EPI	Double	Chain	0.04	0.04	0.05	4.97
(*13*)		Glabridin	EPI-Ant	Single	Ring	0.07	0.03	0.07	4.20
(*14*)		Licoricidin	EPI-Ant	Double	Chain	0.07	0.05	0.06	4.71
(*15*)		Hispaglabridin A	EPI-Ant	Double	Chain and ring	0.05	0.03	0.05	5.24
(*16*)		Hispaglabridin B	Non-EPI	Double	Ring	0.03	0.03	0.04	5.76
**Isoflavene**									
(*17*)		Glabrene	**EPI**	Single	Ring	0.07	0.03	0.07	4.73
**Isoflavone**									
(*18*)		Neobavaisoflavone	**EPI**	Single	Chain	0.07	0.06	0.10	3.30
(*19*)		Wighteone	**EPI**	Single	Chain	0.12	0.12	0.12	2.95
(*20*)		Lupiwighteone	Non-EPI	Single	Chain	0.12	0.07	0.12	2.95
(*21*)		Isowighteone	Non-EPI	Single	Chain	0.12	0.08	0.12	3.02
(*22*)		Glabrone	Non-EPI	Single	Ring	0.07	0.10	0.11	3.47
(*23*)		Licoisoflavone A	**EPI**	Single	Chain	0.15	0.13	0.14	2.68
(*24*)		Licoisoflavone B	EPI-Ant	Single	Ring	0.12	0.13	0.13	3.20
(*25*)		Glycyrrhisoflavone	**EPI**	Single	Chain	0.15	0.14	0.14	2.75
(*26*)		7-*O*-prenylgenistein	Non-EPI	Single	Chain	0.09	0.06	0.10	2.99
(*27*)		8-*C*,7-*O*-diprenylgenistein	Non-EPI	Double	Chain	0.07	0.05	0.08	4.03
**6a-OH-Pterocarpan**									
(*28*)		Glyceollidin II	Non-EPI	Single	Chain	0.10	0.09	0.09	3.25
(*29*)		Glyceollin I	Non-EPI	Single	Ring	0.07	0.08	0.08	3.70
(*30*)		Glyceollin II	Non-EPI	Single	Ring	0.07	0.08	0.08	3.77

(***31***)	Glyceollin III	Non-EPI	Single	Furan	0.07	0.09	0.08	3.18
(***32***)	Glyceollin IV	Non-EPI	Single	Chain	0.06	0.08	0.07	3.52
(***33***)	Glyceollin VI	Non-EPI	Single	Furan	0.04	0.04	0.06	3.63
(***34***)	Glyceofuran	Non-EPI	Single	Furan	0.10	0.14	0.10	3.02
**6a,11a-Pterocarpene**								
(***35***)	Dehydroglyceollin I	EPI-Ant	Single	Ring	0.04	0.02	0.05	4.80
(***36***)	Dehydroglyceollin II	Non-EPI	Single	Ring	0.04	0.03	0.05	4.88
(***37***)	Dehydroglyceollin III	Non-EPI	Single	Furan	0.04	0.02	0.05	4.72

Table S6. The calculation of molecular descriptors obtained from the QSAR model of prenylated (iso)flavonoids as antimicrobials against MRSA vs. as EPIs against *S. aureus* in active EPIs with and without good antimicrobial properties.

	Key descriptor from QSAR as antimicrobials (Kalli *et al*., 2021)								Key descriptors from QSAR as NorA EPIs (this study)			
**Trivial name**	***vsurf_IW7***	***h_pavgQ***	***vsurf_CW3***	***PEOE_VSA_PPOS***	***E_vdw***	***vsurf_DD12***	***PEOE_VSA+2***	***vsurf_D4***	***PEOE_VSA_FPPOS***	***vsurf_HL1***	***PEOE_VSA_FPNEG***	***logP(o/w)***
**EPI with antimicrobial**												
7-*O*-prenylnaringenin (***6***)	0.00	-0.48	1.23	20.65	54.47	0.50	0.00	342.63	0.06	0.08	0.10	3.46
4'-*O*-methylglabridin (***11***)	0.00	-0.01	0.87	10.32	60.47	0.50	0.00	373.75	0.03	0.02	0.05	4.47
Glabridin (***13***)	0.00	-0.01	0.99	20.65	56.09	6.52	0.00	336.25	0.07	0.04	0.07	4.20
Licoricidin (***14***)	0.00	-0.02	0.97	30.97	67.21	0.71	0.00	453.00	0.07	0.05	0.06	4.71
Hispaglabridin A (***15***)	4.08	-0.01	0.88	20.65	65.99	0.50	0.00	431.38	0.05	0.04	0.05	5.24
Licoisoflavone B (***24***)	1.81	-0.68	1.44	39.43	61.98	0.50	0.00	287.50	0.12	0.13	0.13	3.20
Dehydroglyceollin I (***35***)	0.00	-0.01	1.03	10.32	47.39	6.00	16.92	346.50	0.04	0.02	0.05	4.80
**EPI without antimicrobial**												
8-prenylnaringenin (***4***)	0.00	-0.58	1.35	30.97	52.54	0.50	0.00	304.88	0.09	0.08	0.12	3.41
6-*C*,7-*O*-diprenylnaringenin (***7***)	4.97	-0.49	1.03	20.65	63.18	0.50	0.00	407.00	0.05	0.09	0.08	4.49
Glabrene (***17***)	0.00	-0.13	1.04	20.65	55.95	0.50	0.00	332.50	0.07	0.03	0.07	4.73
Neobavaisoflavone (***18***)	0.00	-0.23	1.16	20.65	51.15	0.50	0.00	339.63	0.07	0.06	0.10	3.30
Wighteone (***19***)	3.61	-0.60	1.31	39.43	55.84	0.50	0.00	313.50	0.12	0.12	0.12	2.95
Licoisoflavone A (***23***)	1.51	-0.63	1.44	49.76	59.05	0.71	0.00	304.38	0.15	0.13	0.14	2.68
Glycyrrhisoflavone (***25***)	5.94	-0.64	1.55	49.76	56.94	0.50	16.92	298.88	0.15	0.15	0.14	2.75

Table S7. Structural alignment of prenylated (iso)flavonoids with PQQ16P. The alignment score (S) values were obtained from three repetitions. The highest S values among three repetitions were in put in bold and its overlay structure with PQQ16P was selected as representative.

No.	Compound	Overlay with PQQ16P	S value		
			**1**	**2**	**3**
1.	8-prenylnaringenin (***4***)	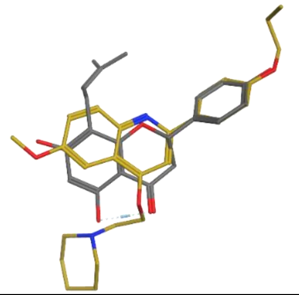	**-68.91**	-64.14	-65.08
2.	6-*C*,7-*O*-diprenylnaringenin (***7***)	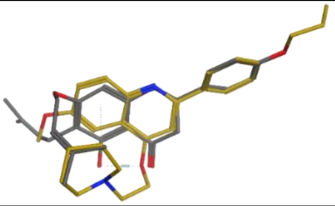	**-74.56**	-72.43	-69.12
3.	Glabrene (***17***)	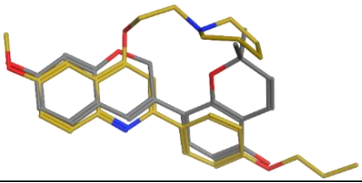	-48.10	-42.90	**-52.25**
4.	Neobavaisoflavone (***18***)	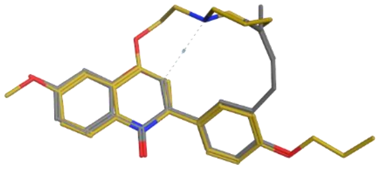	-73,12	-73.34	**-81,50**
5.	Wighteone (***19***)	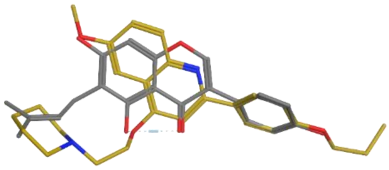	-77.65	**-80.06**	-77.83
6.	Licoisoflavone A (***23***)	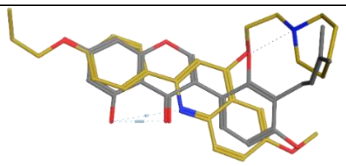	-75.60	-76.12	**-76.94**
7.	Glycyrrhisoflavone (***25***)	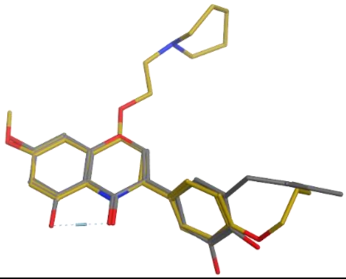	**-72.78**	-71.65	-72.20

References

Kalli, S., Araya-Cloutier, C., Hageman, J., and Vincken, J.-P. Insights into the molecular properties underlying antibacterial activity of prenylated (iso)flavonoids against MRSA. *Sci Rep* (2021) 11, 14180. doi:10.1038/s41598-021-92964-9

Ritsema, J.H., Kramer, N.I., De Bruijn, W.J., Van Dinteren, S., Franssen, M.C., Vincken, J.-P., and Araya-Cloutier, C. Effective membrane permeabilization of methicillin-resistant *Staphylococcus aureus* by prenylated phenolics. *J. Nat. Prod* (2025). doi:10.1021/acs.jnatprod.5c00540

Van De Schans, M.G., Ritschel, T., Bovee, T.F., Sanders, M.G., De Waard, P., Gruppen, H., and Vincken, J.P. Involvement of a hydrophobic pocket and helix 11 in determining the modes of action of prenylated flavonoids and isoflavonoids in the human estrogen receptor. *Chembiochem* (2015) 16, 2668-2677. doi:10.1002/cbic.201500343

Van De Schans, M.G., Vincken, J.-P., De Waard, P., Hamers, A.R., Bovee, T.F., and Gruppen, H. Glyceollins and dehydroglyceollins isolated from soybean act as SERMs and ER subtype-selective phytoestrogens. *J Steroid Biochem Mol Biol* (2016) 156, 53-63. doi:10.1016/j.jsbmb.2015.11.020

Van Dinteren, S., Araya-Cloutier, C., De Bruijn, W.J.C., and Vincken, J.P. A targeted prenylation analysis by a combination of IT-MS and HR-MS: Identification of prenyl number, configuration, and position in different subclasses of (iso)flavonoids. *Analy. Chim. Acta* (2021) 1180, 338874. doi:10.1016/j.aca.2021.338874

Van Dinteren, S., Ritsema, J.H., Sanders, M.G., Meijerink, J., Vincken, J.P., and Araya-Cloutier, C. Unraveling the molecular drivers of antibacterial prenylated (iso)flavonoids and chalcones against *Streptococcus mutans*. *Sci Rep* (2025) 15, 14776. doi:10.1038/s41598-025-98782-7
